# Iron-sulphur cluster biogenesis factor LYRM4 is a novel prognostic biomarker associated with immune infiltrates in hepatocellular carcinoma

**DOI:** 10.1186/s12935-021-02131-3

**Published:** 2021-09-06

**Authors:** Yilin Pang, Guoqiang Tan, Xunjun Yang, Yuanshan Lin, Yao Chen, Jinping Zhang, Ting Xie, Huaibin Zhou, Jun Fang, Qiongya Zhao, Xiaojun Ren, Jianghui Li, Jianxin Lyu, Zheng Wang

**Affiliations:** 1 College of Bioscience and Biotechnology, Hunan Agricultural University, Changsha, 410128 Hunan China; 2 Zhejiang Provincial Key Laboratory of Medical Genetics, Key Laboratory of Laboratory Medicine, Ministry of Education, China, School of Laboratory Medicine and Life Sciences, Wenzhou Medical University, Wenzhou, 325035 Zhejiang China; 3 Department of Laboratory Medicine, The Second Affiliated Hospital and Yuying Children’s Hospital of Wenzhou Medical University, Wenzhou, 325027 Zhejiang China; 4 People’s Hospital of Hangzhou Medical College, Hangzhou, 310014 China; 5 Department of Pathology, The First Affiliated Hospital of Wenzhou Medical University, Wenzhou, China

**Keywords:** LYRM4, Hepatocellular carcinoma, Prognosis, Diagnostic biomarker, Functional network analysis

## Abstract

**Background:**

LYRM4 is necessary to maintain the stability and activity of the human cysteine desulfurase complex NFS1-LYRM4-ACP. The existing experimental results indicate that cancer cells rely on the high expression of NFS1. However, the role of LYRM4 in liver hepatocellular carcinoma (LIHC) remains unclear.

**Methods:**

In this study, we combined bioinformatics analysis and clinical specimens to evaluate the mRNA, protein expression, and gene regulatory network of LYRM4 in LIHC. Furthermore, we detected the activity of several classical iron-sulphur proteins in LIHC cell lines through UV-vis spectrophotometry.

**Results:**

The mRNA and protein levels of LYRM4 were upregulated in LIHC. Subsequent analysis revealed that the *LYRM4* mRNA expression was related to various clinical stratifications, prognosis, and survival of LIHC patients. In addition, the mRNA expression of *LYRM4* was significantly associated with ALT, tumour thrombus, and encapsulation of HBV-related LIHC patients. IHC results confirmed that LYRM4 was highly expressed in LIHC tissues and showed that the expression of LYRM4 protein in LIHC was significantly correlated with age and serum low-density lipoprotein (LDL) and triglyceride (TG) content. In particular, the mRNA expression of key iron- sulphur proteins POLD1 and PRIM2 was significantly overexpressed and correlated with poor prognosis in LIHC patients. Compared with hepatocytes, the activities of mitochondrial complex I and aconitate hydratase (ACO2) in LIHC cell lines were significantly increased. These results indicated that the iron-sulphur cluster (ISC) biosynthesis was significantly elevated in LIHC, leading to ISC-dependent metabolic reprogramming. Changes in the activity of ISC-dependent proteins may also occur in paracancerous tissues. Further analysis of the biological interaction and gene regulation networks of LYRM4 suggested that these genes were mainly involved in the citric acid cycle and oxidative phosphorylation. Finally, LYRM4 expression in LIHC was significantly positively correlated with the infiltrating levels of six immune cell types, and both factors were strongly associated with prognosis.

**Conclusion:**

LYRM4 could be a novel prognostic biomarker and molecular target for LIHC therapy. In particular, the potential regulatory networks of LYRM4 overexpression in LIHC provide a scientific basis for future research on the role of the ISC assembly mechanism and LYRM4-mediated sulphur transfer routes in carcinogenesis.

**Supplementary Information:**

The online version contains supplementary material available at 10.1186/s12935-021-02131-3.

## Background

LIHC is the fifth most common malignancy and the second most frequent cause of cancer-related mortality worldwide [1, 2]. Recent studies from several countries have reported a significant age-specific increase in LIHC development among people over 75 years old [2, 3]. Based on annual projections, the World Health Organization predicts that more than 1 million patients with LIHC will die by 2030 [4]. LIHC accounts for approximately 85%–90% of all primary liver malignancies. The rising incidence of LIHC is correlated with the increasing prevalence of hepatitis B virus (HBV), hepatitis C virus (HCV) infections and alcohol abuse, particularly in East Asia [5, 6]. Although therapies with sorafenib and regorafenib provide a modest survival benefit, overall anti-tumor responses are limited [7], with a 5-year survival rate of only 18% for LIHC [8]. Therefore, it is necessary to screen novel biomarkers for early diagnosis and identify new therapeutic targets for LIHC by screening the changes in the gene function network related to the formation and progression of LIHC.

Interestingly, traditional methods focus more on the LIHC itself to stratify and screen prognostic gene sets. In contrast, Gong et al. [9] developed a new method to identify LIHC subtypes and prognostic gene sets based on the activity changes of immunologic and hallmark gene sets in tumour and non-tumour tissues, since the activity changes of these gene sets in adjacent non-tumour tissues may substantially impact prognosis by affecting the proliferation of the remaining liver cells and colonisation of circulating tumour cells after treatment measures such as hepatectomy, so as to improve patient outcomes with personalised medicine and accurate assessment of prognosis.

Iron-sulphur (Fe-S) clusters are ubiquitous in all life forms. They have become integral parts of many physiological functions, including energy conversion, protein translation, DNA replication, and repair [10]. The human mitochondrial cysteine desulfurase complex consists of a catalytic subunit (NFS1), an LYR protein (LYRM4, which is also an alternative name to ISD11), and an acyl carrier protein (ACP), in which the C-terminus of LYRM4 is essential for forming a stable and active complex with NFS1 [11–13]. The cysteine desulfurase complex further interacts with mitochondrial scaffold protein ISCU, frataxin (FXN) (is a kinetic activator of persulfide transfer) and ferredoxin FDX2 (allows persulfide reduction into sulphide), forming the “core ISC complex” for *de novo* Fe-S cluster synthesis [14]. It also acts as a sulphur donor for other processes, such as tRNA synthesis [15–17] and molybdenum cofactor biosynthesis [17, 18]. In addition, the LYR superfamily forms lock-and-key interactions with acyl-ACP, which is associated with fatty acid biosynthesis to coordinate the expression, Fe/S cofactor maturation, and activity of the respiratory complexes [19–21].

A recent report showed that NFS1 is overexpressed in most well-differentiated lung adenocarcinomas because incipient lung tumours survive in a high oxygen environment. NFS1 activity is particularly important for maintaining iron-sulphur co-factors present in at least 48 human cell-essential enzymes. Thus, overexpression of NFS1 in adenocarcinomas leads to resistance to high oxygen tension and protects cells from undergoing ferroptosis in response to oxidative damage [22]. Consistent with this, our analysis of RNA-Seq data from 159 HBV-related LIHC patients showed that *NFS1* mRNA was overexpressed in tumour tissues compared to paired adjacent liver tissues (*p* ˂ 0.05) [5]. Shi et al. [23] used RNA-interference techniques to demonstrate that human LYRM4 is essential for both ISC biogenesis and maintenance of normal cellular iron homeostasis in HeLa cells. However, there have been no reports on the differential expression of NFS1 and its auxiliary protein LYRM4 in tumour tissues.

Liver cells contain approximately two thousand mitochondria, which are the energy and metabolic factories of cells [24]. Therefore, it is necessary to evaluate the expression of LYRM4 in pan-cancer and to investigate whether abnormal expression of LYRM4 is closely related to the tumorigenesis and development of LIHC. In the present study, we investigated the expression profile, prognostic significance, and gene regulation network of LYRM4 in LIHC based on different open databases by utilizing multi-dimensional analysis methods. Moreover, we used IHC to detect the protein expression level of LYRM4 in LIHC and paired adjacent normal tissues and analysed the correlation between their characteristics. In addition, co-expression analysis was performed between *LYRM4* and the genes involved in mitochondrial ISC biosynthesis in tumour and non-tumour tissues. The changes in ISC-dependent key iron-sulphur proteins and their relationship with the prognosis of LIHC patients were also analysed. Finally, we explored the correlation between LYRM4 expression and immune infiltration. These findings will aid in revealing the potential pathogenesis mechanism of LYRM4 in LIHC and show that LYRM4 may be a useful biomarker and target in the treatment of LIHC.

## Methods

### Reagents and antibodies

Horseradish peroxidase (HRP)-conjugated anti-rabbit and anti-mouse immunoglobulin G, and monoclonal antibody against β-Actin were obtained from Beyotime (Shanghai, China). BCA protein assay kit, SuperSignal West Femto maximum sensitivity substrate, and pierce protease and phosphatase inhibitor mini tablets, EDTA-free were obtained from Thermo Scientific (Waltham, MA). Rabbit polyclonal to LYRM4 was purchased from Abcam (Cambridge, UK). Streptavidin-biotin-peroxidase complex (SABC) and 3,3′-Diaminobenzidine tetrahydrochloride (DAB) were purchased from ZSGB-BIO (Beijing, China).

### Tissue microarrays (TMA) and IHC staining

We collected paraffin tissue specimens from 92 patients diagnosed with LIHC from the First Affiliated Hospital of Wenzhou Medical University from May 2018 to October 2019, and detailed clinical data and laboratory examination results of all patients were tracked and recorded. The inclusion criteria were as follows: (1) the pathologic diagnosis was LIHC; (2) no chemotherapy or radiation therapy was administered before surgery; (3) detailed hospitalisation information; and (4) patients who volunteered to participate in this study and had lived in Wenzhou for more than 5 years. The exclusion criteria were as follows: (1) basic metabolic and infectious diseases; (2) history of other tumours; (3) radiotherapy and/or chemotherapy; and (4) inability to obtain complete clinical data.

All collected formalin-fixed, paraffin-embedded LIHC tissues were prepared to construct TMA, as described in a previous study [25]. IHC staining was performed following previously described protocols, with minor modifications [26]. The TMA slides were incubated at 4 °C overnight with primary antibodies against LYRM4 (1:100) or PBS as a negative control, followed by incubation with biotin-labelled goat anti-rabbit IgG at 37 °C for 30 min, and then incubated with SABC at room temperature for 30 min. Signals were visualised with DAB, counterstained with hematoxylin, dehydrated in ethanol, cleared in xylene, and then fixed.

### Evaluation of IHC staining

IHC images were reviewed and scored independently by two pathologists who had no prior knowledge of the clinicopathological features of the TMA specimens. The relative protein expression of LYRM4 was analysed by calculating the integrated optical density per stained area (IOD/area, mean OD value, MOD) using Image-Pro Plus 6.0 (Media Cybernetics, Rockville, MD, USA) according to a previously described protocol [27]. Specific scoring criteria were as follows: H-score < 7 was categorised as the low expression group, and H-score ≥ 7 was categorised as the high expression group.

### Cell culture

The human LIHC cell lines HCCLM3, MHCC97H, HepG2 and hepatocytes L-02 cells were obtained from the Cell Bank of the Chinese Academy of Sciences (Shanghai, China). All cells were cultured in high-glucose DMEM (GIBCO, Waltham, MA, USA) containing 10% FBS (fetal bovine serum) (GIBCO, USA) and antibiotics (100 U/ml penicillin and 100 μg/ml streptomycin) (GIBCO, USA), incubated at 37 °C in a humidified incubator with 5% CO_2_. Cell lines were routinely tested and confirmed to be mycoplasma free during this study.

### Western blot analysis

Proteins from whole-cell was extracted with 1% Triton X-100 lysis buffer supplemented with protease and phosphatase inhibitors. Protein concentrations of the extracts were determined by the BCA assay kit. 40 μg total protein from each sample was separated by ExpressPlus PAGE Gel (10 × 8, 4–20%, 15 wells) (Genscript, Nanjing, China) and transferred onto 0.22 µm PVDF membrane (Bio-Rad, Hercules, CA, USA) using a semidry transfer system (Bio-Rad, USA). Blots were blocked at room temperature for 15 min in QuickBlock Blocking Buffer for Western Blot (Beyotime, Shanghai, China) on a shaker, and then incubated with primary antibodies specific to LYRM4 (1:2000) and β-actin (1:5000) overnight at 4 ºC, respectively. The membrane was washed in TBST for 3 × 10 min and then incubated with HRP-conjugated anti-rabbit (1:5000) and anti-mouse (1:20000) IgG secondary antibody at room temperature for 1.5 h while gently agitating. Signals were detected with SuperSignal West Femto maximum sensitivity substrate according to the manufacturer`s protocol.

### Isolation and purification of mitochondria

A mitochondria extraction kit (Solarbio, Beijing, China) was used to isolate and purify the mitochondria as described previously [28, 29].

### Mitochondrial complex I activity assay

Complex I activity was measured as previously described [30, 31].

### Mitochondrial aconitate hydratase activity assay

The prepared mitochondrial protein (5 µL; 2 µg/µL) was added to the assay buffer containing Tris (pH 8.0; 100 mM), cis-aconitic acid (1 mM), MgCl_2_ (5 mM), and isocitrate dehydrogenase (4 U/ml). The mixture was incubated at 37 °C for 2 min. After reading the baseline at 340 nm, NADP^+^ was added, and the increase in absorbance was recorded at 340 nm for 80 s. The enzymatic activity of each mitochondrial enzyme was corrected using citrate synthase (CS) activity.

### Oncomine database analysis

The Oncomine 4.5 database (https://www.oncomine.org/resource/login.html) is the largest oncogene chip database and integrated data mining platform, including 715 gene expression datasets and clinical data from 86,733 cancer tissues and normal tissues [32]. The retrieval condition was set as: Analysis Type/Cancer vs. Normal Analysis, Cancer Type/Liver Cancer, Dataset Filters/Data Type/mRNA or DNA, Sample Filters/Sample Type/Clinical Specime. Then, the mRNA expression and DNA copy number of *LYRM4* in LIHC were analyzed by the Oncomine database. The results are displayed with the fold change over 2. Student's *t*-test was used to analyze differences in the expression of *LYRM4* between normal controls and cancer specimens. A cutoff of *p* < 0.05 was considered statistically significant.

### GEPIA2 analysis

The GEPIA2 database (http://gepia2.cancer-pku.cn/#index), developed by Peking University in China, is based on TCGA and GTEx database which includes RNA sequencing and expression data from 33 malignant tumour, 8587 normal tissue, and 9736 tumour samples [33]. The expression differences of *LYRM4* between tumours and normal tissues in distinct types of cancer were determined within the GEPIA2 database. We also used GEPIA2 to analyze the correlation between *LYRM4* expression and pathologic staging and survival of LIHC patients, including Overall Survival and Disease Free Survival, in LIHC. The *p*, *p*(HR) or Pr(> F) values from a log-rank test were included in the plot.

### UALCAN database analysis

UALCAN (http://ualcan.path.uab.edu/) is a comprehensive and user-friendly web resource to perform in-depth analyses of TCGA gene expression data [34]. In this study, we used UALCAN to analyze the expression differences of *LYRM4* and its promoter methylation in LIHC, as well as in various tumor subgroups based on age, individual cancer stages, tumor grade and other clinical stratified criteria. Student’s *t*-test was used to generate *p*-values; after Bonferroni correction for multiple measures, *p* was still < 0.05, which was statistically significant.

### STRING analysis

The String database (https://string-db.org/) is a web-based platform for analyzing the interactions between genes or proteins, including direct physical interactions between proteins and indirect functional correlations between proteins [35]. It integrates experimental data, abstracts from PubMed and data from other databases. In addition, it provides bioinformatics-based predictions. We used STRING to search the network map of proteins associated with LYRM4. The database key setting parameter was set to medium confidence 0.4 and the maximal number of interactors was 50.

### GeneMANIA analysis

GeneMANIA (http://genemania.org/) is a commonly used interactive website for constructing PPI networks and predicting the function of genes. The website features several bioinformatic methods, including gene co-expression, co-localization, pathway, physical interactions and predicted [36]. We used GeneMANIA to construct the gene networks and predict the function of LYRM4 and enriched genes in LIHC identified by GSEA (miRNA 495 and transcription factor GGAANCGGAANY_UNKNOWN).

### LinkedOmics analysis

The LinkedOmics database (http://www.linkedomics.org/login.php) is a comprehensive and user-friendly web interface with integrated multi-omics data and clinical data from 11,158 patients (32 cancer types) from TCGA project [37]. In this study, LinkedOmics was utilized to study genes differentially expressed in correlation with *LYRM4* in LIHC. Pearson’s correlation coefficient was applied in statistical analysis of the result produced by LinkedOmics. GSEA was utilized to perform various enrichment analyses, including GO, KEGG pathways, kinase-target, miRNA-target and transcription factor-target. The result of LinkFinder was ranked based on criteria of FDR < 0.05 and 500 simulations.

### TargetScan analysis

TargetScan (http://www.targetscan.org/vert_72/) is a flexible, user-friendly web interface that predicts biological targets of miRNAs by searching for the presence of conserved 8mer, 7mer, and 6mer sites that match the seed region of each miRNA [38]. We used TargetScan to analyse miRNAs differentially expressed in connection with *LYRM4* and predict the biological target genes of these miRNAs.

### FunRich analysis

FunRich (http://www.funrich.org/) is an independent programming tool for functional enrichment and interaction network analysis of genes and proteins [39]. In this study, we used Funrich tool for the gene sets that were enriched in the target network of *LYRM4* and miRNA enrichment analysis, including Biological process, Cellular component, Molecular function and Biological pathway.

### TIMER2.0 analysis

TIMER2.0 (http://timer.cistrome.org/) is a comprehensive resource for the systematic investigation of immune infiltrates across diverse cancer types [40]. We first analysed the correlations between the expression of *LYRM4* and the abundance of the six immune cell types in LIHC using Spearman tests (tumour purity adjusted). The survival module was used to draw Kaplan-Meier plots for immune infiltrates and *LYRM4* to determine survival differences. Finally, the expression of *LYRM4* in various tumours was analysed using the TIMER database, and the results were analysed statistically using the Wilcoxon rank sum test. Statistical significance was set at *p* < 0.05.

### Statistical analysis

All statistical analyses were performed using SPSS 21.0 (IBM, Armonk, NY, USA). Data conforming to a normal distribution are presented as mean ± SD. Comparisons between two groups were performed using an independent Student’s *t*-test. Measurement data that did not conform to the normal distribution were represented by M (P25, P75), and the comparison between the two groups was performed using the rank sum test in the IHC results. The χ^2^ test was performed to evaluate the relationship between clinicopathological features and LYRM4 expression in the IHC results. The Wilcoxon signed-rank test was used to analyse the significance of protein and mRNA expression in adjacent normal and cancer tissues of LIHC and HBV-Related LIHC. Statistical significance was set at *p* < 0.05.

## Results

### LYRM4 expression and its correlation with clinicopathological parameters of LIHC

**Fig. 1:**
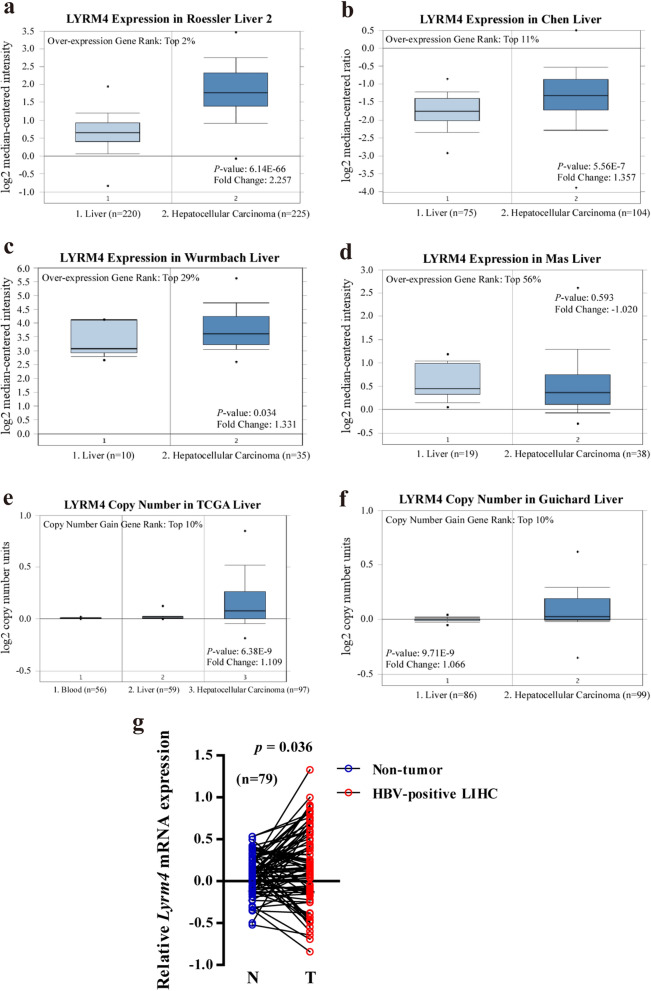
Expression levels of LYRM4 mRNA and DNA copy number in LIHC tissue samples. **a**–**d** Box plots showing *LYRM4* mRNA expression levels in Roessler Liver 2, Chen Liver, Wurmbach Liver, and Mas Liver datasets, respectively (Oncomine database). **e**–**f** Box plots showing *LYRM4* copy number in The Cancer Genome Atlas (TCGA) Liver and Guichard Liver datasets, respectively. The fold change, associated *p* values, and overexpression gene rank were based on Oncomine analysis. **g** The mRNA expression of *LYRM4* in HBV-related LIHC and paired non-tumour liver tissues was investigated using RNA-seq (n = 79). These figure results were obtained from the studies of Gao et al. [5], and all the LIHC patients are over 54 years old. The top and bottom black dots in the figure represent the maximum and minimum values of samples, respectively

To determine differences in *LYRM4* mRNA expression in tumour and paired normal tissues, the *LYRM4* gene expression profile across diverse cancer types was determined using the Oncomine, UALCAN, and GEPIA2 databases. *LYRM4* expression was higher in lymphoid neoplasms, such as diffuse large B-cell lymphoma (DLBC), leukaemia, sarcoma, thymoma (THYM), LIHC, colorectal cancer, gastric cancer, lung cancer, melanoma, and lymphoma, compared to normal samples. The mRNA level of *LYRM4* was significantly decreased in kidney chromophobe (KICH), brain, and CNS cancer tissues compared with matched normal tissues (Additional file 1: Figure S1a–b). The difference in *LYRM4* expression in LIHC was further evaluated using the Oncomine and GEPIA2 databases. The results showed that the mRNA expression and DNA copy number variation (CNV) of *LYRM4* were significantly higher in LIHC tissues than in normal tissues (*p* ˂ 0.05) (Fig. 1a-c, e, f and Additional file 1: Figure S1c). Consistently, *LYRM4* mRNA levels were also significantly upregulated in HBV-related LIHC patients older than 54 years (Fig. 1g) [5].

**Fig. 2:**
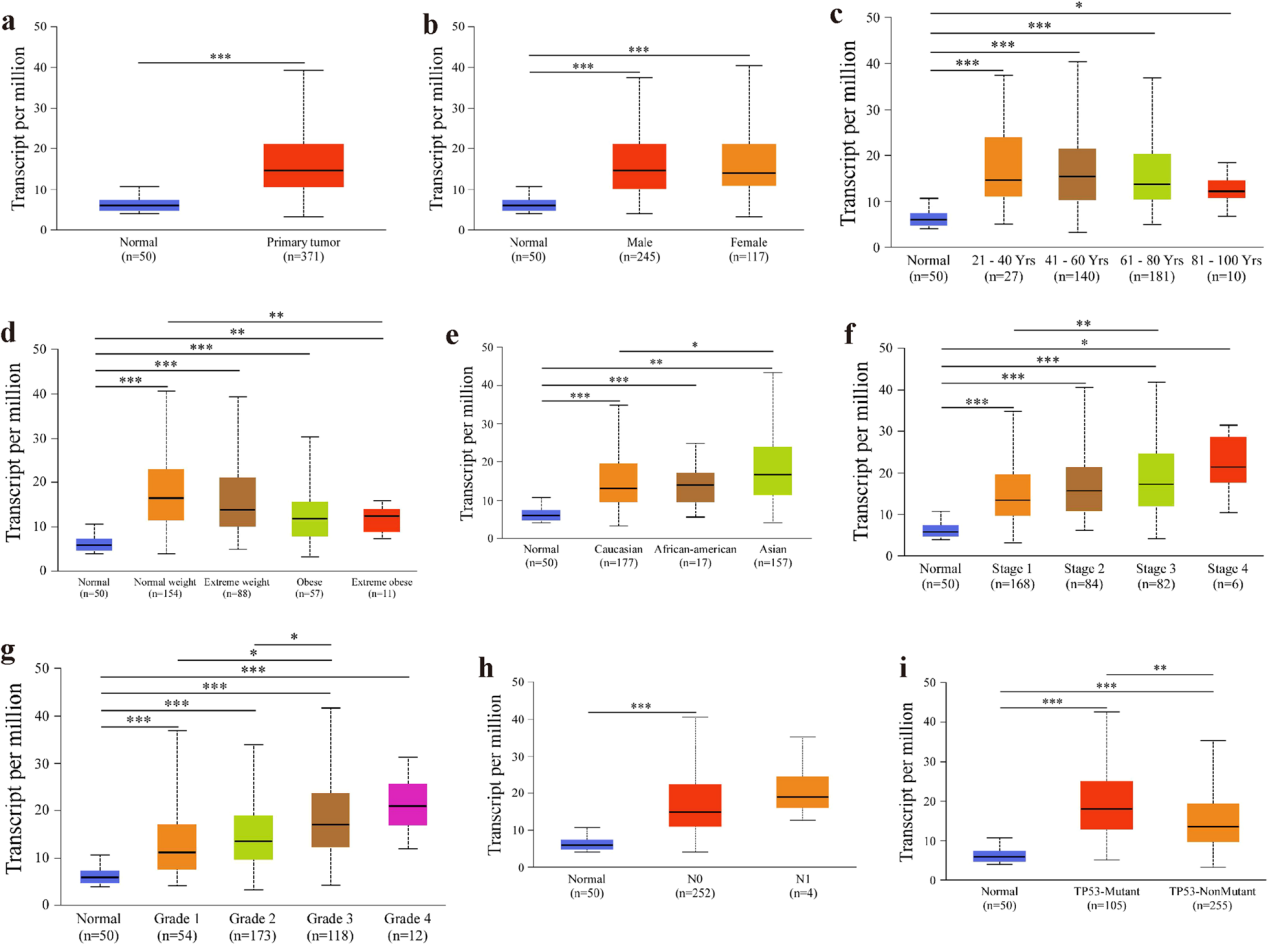
Relative expression of *LYRM4* mRNA in subgroups of patients with LIHC, classified according to sample types (**a**)**,** sex (**b**), age (**c**), weight (**d**), race (**e**), individual cancer stages (**f**), tumour grade (**g**), nodal metastasis status (**h**), and TP53 mutation status (**i**) (UALCAN database). Data are shown as mean ± SE. *, *p* < 0.05; **, *p* < 0.01; ***, *p* < 0.001. The asterisk indicates a significant difference between the two sets of data

Further subgroup analysis of multiple clinicopathological features of *LYRM4* in LIHC reliably indicated high levels of *LYRM4* transcription. The transcription level of *LYRM4* was significantly higher in LIHC patients than that in healthy people in subgroup analyses based on age (normal vs. age [21–40 y], normal vs. age [41–60 y], normal vs. age [61–80 y], and normal vs. age [81-100 y], *p* ˂ 0.001). Moreover, in the sex, weight, race, individual cancer stage, tumour grade, nodal metastasis, and TP53 mutation status subgroup analyses, the expression of *LYRM4* mRNA was also significantly higher in LIHC patients (Fig. 2).

In addition, we analysed the association between *LYRM4* mRNA expression and various clinicopathological factors of HBV-related LIHC using paired tumours and adjacent liver tissues from 157 patients (Additional file 2: Table S1) [5]. As shown in Table 1, Pearson chi-square test analyses revealed that the mRNA expression of *LYRM4* was significantly associated with tumour thrombus (*p* ˂ 0.05). Multivariate analysis showed that the mRNA expression of *LYRM4* was related to ALT (glutamic-pyruvic transaminase), tumour thrombus, and encapsulation of HBV-related LIHC patients (*p* ˂ 0.05).

**Table 1:** Association between *LYRM4* mRNA expression and various clinicopathological factors of HBV-related LIHC patients

Variables	*LYRM4* mRNA expression		*p* value	Multivariate analysis^a^		
	Low (n = 79)	High (n = 78)		OR	95%	*p* value
Age, years						
≤ 54	48	37	0.09	1	Reference	
> 54	31	41		1.570	0.749–3.288	0.232
Gender						
Male	62	65	0.44	1	Reference	
Female	17	13		0.700	0.271–1.806	0.461
Preoperative AFP (ng/ml)						
≤ 200	38	49	0.06	1	Reference	
> 200	40	28		0.675	0.312–1.461	0.318
TB (µmol/L)						
≤ 20	77	73	0.43	1	Reference	
> 20	2	5		2.995	0.457–19.63	0.253
ALT (U/L)						
≤ 50	50	60	0.91	1	Reference	
> 50	29	36		0.405	0.170–0.968	**0.042**
γ-GT (U/L)						
≤ 60	41	40	1.00	1	Reference	
> 60	38	37		1.957	0.830–4.615	0.125
HBcAb						
No	5	4	0.98	1	Reference	
Yes	74	74		1.779	0.356–8.877	0.483
Tumor size (cm)						
≤ 5	39	36	0.69	1	Reference	
> 5	40	42		1.382	0.612–3.124	0.436
Tumor number						
Single	58	58	0.89	1	Reference	
Multiple	21	20		1.226	0.523–2.871	0.639
Tumor thrombus						
No	55	66	**0.03**	1	Reference	
Yes	24	12		0.355	0.132–0.957	**0.041**
Tumor encapsulation						
No	27	17	0.08	1	Reference	
Yes	50	59		2.345	1.035–5.315	**0.041**
Tumor differentiation						
High	37	45	0.15	1	Reference	
Low	42	32		0.771	0.368–1.618	0.492
History of liver cirrhosis						
No	19	27	0.15	1	Reference	
Yes	60	51		0.519	0.223–1.212	0.130

Pearson Chi-Square test was used to analyze the relationship between *LYRM4* mRNA expression and clinicopathological characteristics. Font bold means have statistical significance *p* < 0.05

^a^Logistics regression was used to multivariate analysis

### Promoter methylation levels of *LYRM4* in LIHC

**Fig. 3:**
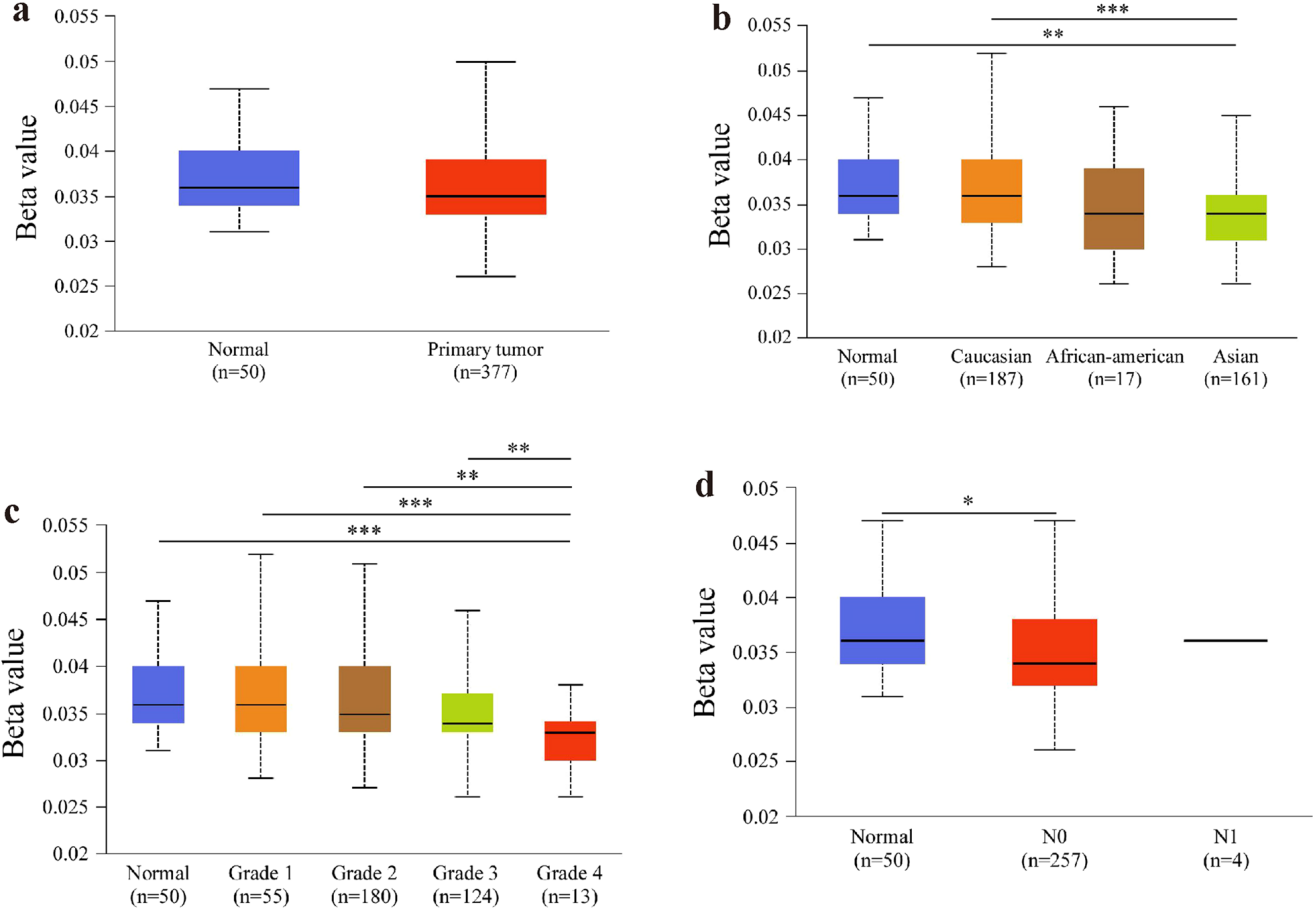
*LYRM4* promoter methylation profile of LIHC patient subgroups based on sample types (**a**), race (**b**), tumour grade (**c**), and nodal metastasis status (**d**) (UALCAN database). Data are shown as mean ± SE. *, *p* < 0.05; **, *p* < 0.01; ***, *p* < 0.001. The asterisk indicates a significant difference between the two sets of data

To evaluate the promoter methylation levels of *LYRM4* in LIHC, we used the UALCAN database to analyse the correlation between *LYRM4* expression and promoter DNA methylation. No statistical difference in the promoter methylation level of *LYRM4* was observed in LIHC tissues compared to normal tissues. However, further subgroup analysis of the LYRM4 promoter methylation profile revealed great significance according to race, tumour grade, and nodal metastasis status in LIHC patients (Fig. 3).

### Prognostic potential of *LYRM4* in LIHC

**Fig. 4:**
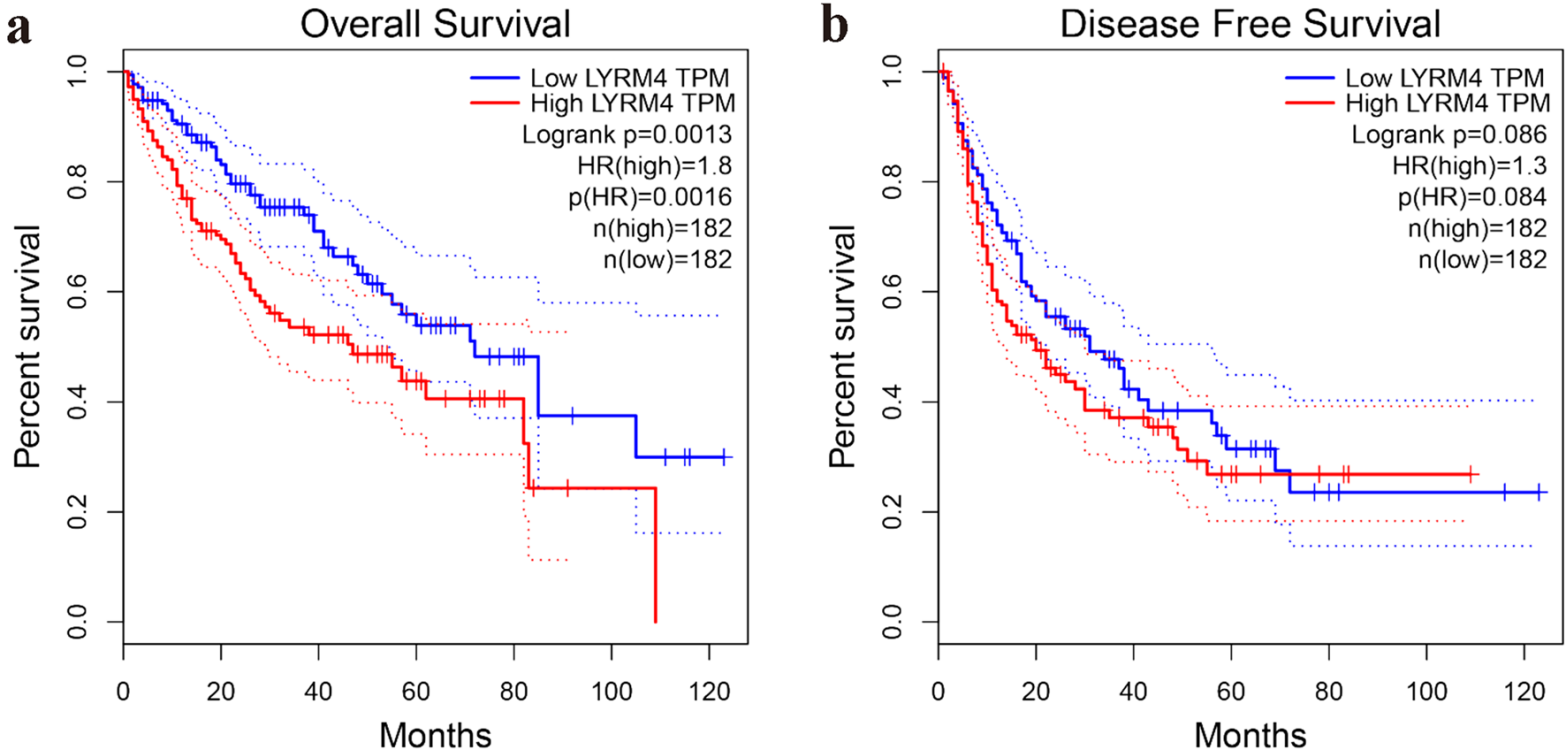
*LYRM4* is a poor prognostic factor for LIHC progression and patient survival (GEPIA2 database). **a** Kaplan-Meier overall survival (OS) curve of LIHC patients (n = 364) based on *LYRM4* mRNA expression level. **b** Kaplan-Meier disease free survival analysis was performed on LIHC patients according to the expression level of *LYRM4*

To further assess whether high *LYRM4* mRNA expression levels are associated with clinical and pathologic parameters of LIHC patients, we used the GEPIA2 database to analyse the prognostic value of *LYRM4* in LIHC. According to the Kaplan-Meier survival analysis, LIHC patients with high *LYRM4* expression had lower overall survival rates than those with low *LYRM4* expression (Fig. 4a, *p* < 0.01). However, *LYRM4* mRNA expression seemed to have little impact on the disease/progression-free survival of LIHC patients (Fig. 4b).

### Elevated protein expression of LYRM4 contributes to reprogramming of iron-sulphur proteins-related metabolic pathways in LIHC cells

**Fig. 5:**
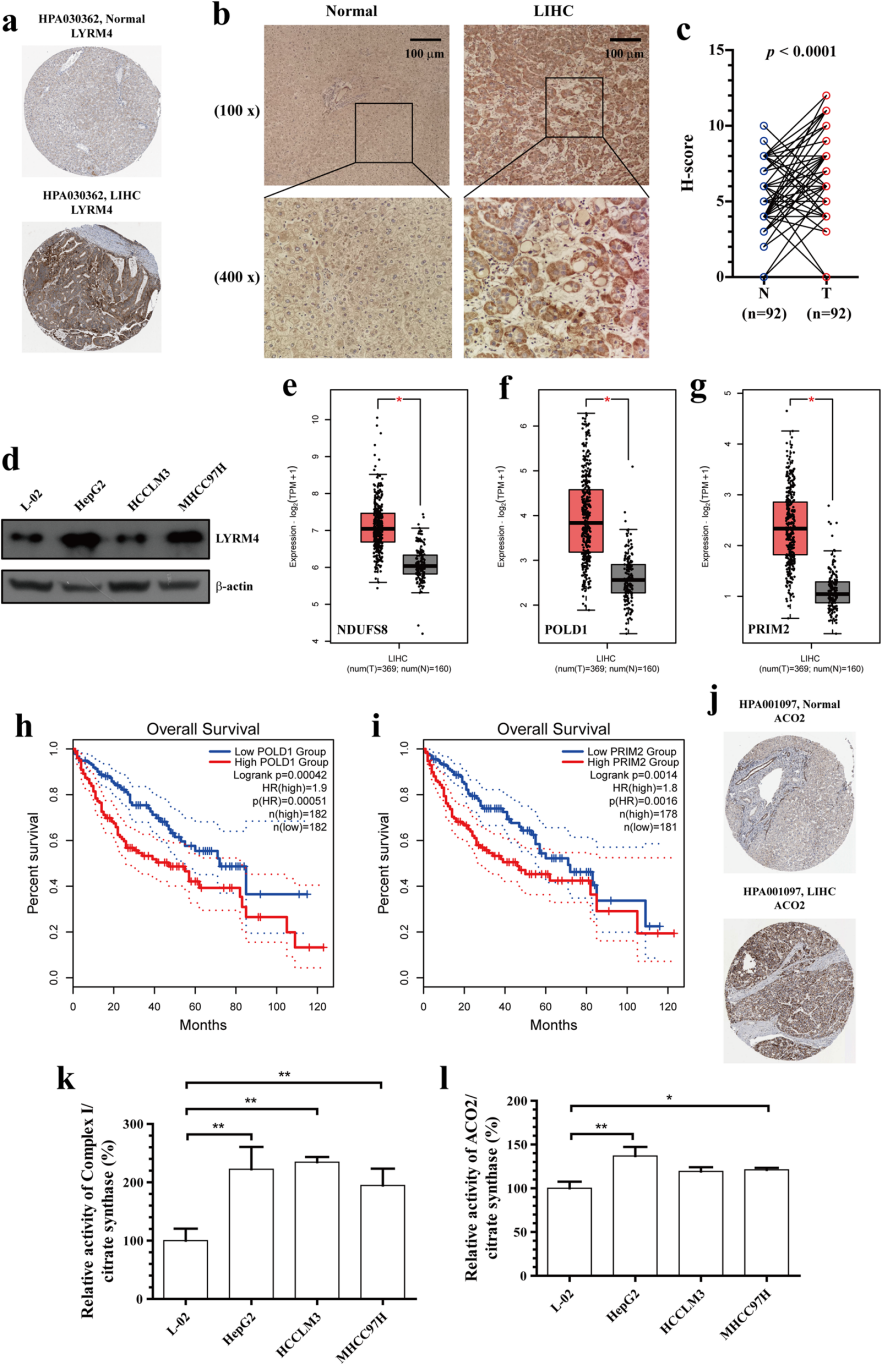
Protein expression of LYRM4 and Fe-S cluster biosynthesis were significantly increased in LIHC and affected LIHC patient prognosis. **a** The protein expression levels of LYRM4 were elevated in LIHC (The Human Protein Atlas database). **b** Representative immunohistochemistry images of LYRM4 in tissue arrays containing human LIHC specimens and matched adjacent normal tissues. Regions in squares are magnified 4× in bottom panels. **c** Summary statistics of H-score based on only intact and paired specimens (n = 92). **d** Western blot analysis of LYRM4 in normal hepatocytes and three LIHC cell lines. **e**–**g** Box plots showing *NDUFS8* (**e**), *POLD1* (**f**), and *PRIM2* (**g**) mRNA expression levels in LIHC (GEPIA2). **h**–**i** Kaplan-Meier diagram of the relationship between *POLD1* (**h**) and *PRIM2* (**i**) gene expression and survival in LIHC patients (GEPIA2). **j** Protein expression of mitochondrial aconitase ACO2 in LIHC increased (The Human Protein Atlas database). **k**–**l** Activities of mitochondrial complex I and ACO2 were detected in the human LIHC cell lines and hepatocytes, and the histogram represented the relative activity of control (hepatocytes). Data are presented as means ± SD (n = 3). *, *p* < 0.05; **, *p* < 0.01

To further explore the clinical significance of LYRM4 in LIHC, LYRM4 protein expression was analysed using the Human Protein Atlas database. As shown in Fig. 5a, LYRM4 was not detected in the normal liver and showed weak to medium staining in LIHC. To increase the credibility of the results, IHC staining was performed in TMA containing 92 archived paraffin-embedded LIHC specimens (Additional file 3: Figure S2–4). Consistently, protein analysis on TMA slides confirmed that LYRM4 was dramatically overexpressed in LIHC tissues compared to adjacent normal tissues (Fig. 5b-c). The association between LYRM4 protein expression and clinicopathological features of LIHC was analysed using the chi-square test or rank sum test (Additional file 4: Table S2). As shown in Table 2, these results suggested that the expression of LYRM4 was significantly associated with age (*p* = 0.0410), serum LDL (*p* = 0.0340), and TG content (*p* = 0.0130). No significant relationship was found between LYRM4 protein expression and variables such as alcohol consumption. However, drinking habits dramatically increased the expression level (mean) of LYRM4 in both LIHC and paracancerous tissues (Additional file 5: Figure S14). Additionally, we found increased LYRM4 in both HBV-related (MHCC97H) and negative (HepG2) LIHC cell lines (Fig. 5d and Additional file 3: Figure S5) compared to that in hepatocytes L-02 cells.

**Table 2:** Association between LYRM4 protein expression and various clinicopathological factors in LIHC patients

Variables	LYRM4 protein expression		*p* value
	Low (n = 43)	High (n = 49)	
Gender			
Male	36	41	0.9951
Female	7	8	
History of drinking			
Yes	12	20	0.2226
No	30	29	
Hepatitis B			
Yes	30	41	0.7605
No	2	2	
History of smoking			
Yes	17	20	0.8363
No	26	28	
Liver cirrhosis			
Yes	33	31	0.1610
No	10	18	
History of Hepatitis			
Yes	28	34	0.6628
No	15	15	
Grade (G)			
G1–G2	37	41	0.8785
G3	5	5	
TNM stage			
I–II	31	33	0.6216
III–IV	12	16	
Tumor thrombus			
Yes	11	15	0.5973
No	31	33	
Tumor encapsulation			
Yes	6	4	0.2790
No	31	43	
Tumor size	3.65 (2.2, 6.13)	3 (2.0, 5)	0.1850
Age	59 ± 10.9	55 ± 8.4	**0.0410**
ALB (g/L)	35.9 ± 5.1	37.1 ± 4.8	0.2240
LDL (mmol/L)	2.0 ± 0.7	2.3 ± 0.8	**0.0340**
AFP (ng/mL)	16.7 (3.9, 291.7)	32.2 (4.7, 388.3)	0.5340
TBIL (mmol/L)	13 (11, 21.5)	12 (10, 17)	0.4100
DBIL (mmol/L)	7 (5, 10.3)	6 (4.5, 9)	0.3030
IBIL (mmol/L)	6 (5, 10)	6 (5, 9)	0.6600
ALT (U/L)	53.5 (30.8, 116)	52 (36, 86.5)	0.8750
AST (U/L)	37.5 (27, 53.5)	34 (23.5, 57.5)	0.5650
ALP (U/L)	89.5 (73.8, 122.3)	94 (75.5, 118)	0.8600
TG (mmol/L)	0.9 (0.7, 1.1)	1.1 (0.9, 1.5)	**0.0130**

The bold values indicate the significant difference between the two groups of data, and the statistical significance was set at *p* < 0.05

It is well known that ISC assembly proteins are encoded by the gene cluster *iscSUA-hscBA-fdx* in *Escherichia coli* and are highly conserved among aerobic organisms from bacteria to humans [41]. To determine whether similar transcriptional mechanisms operate in LIHC cells, a co-expression analysis was performed between LYRM4 and the 19 proteins involved in mitochondrial Fe/S protein assembly and export at transcript levels in LIHC using the GEPIA2 database. As shown in Table 3, in the liver, the expression of the LYRM4 gene was significantly positively correlated with the expression of 16 other genes, excluding NFS1, NUBPL, and MFRN2, among which the co-expression correlation coefficient (R) of four genes (*NFU1*, *ISCU*, *ISCA1*, and *ACP1*) were all greater than 0.7 (*p* = 0). However, in LIHC normal tissues and LIHC tumour tissues, the co-expression correlation between *LYRM4* and these genes was sequentially reduced, or even negatively correlated.

**Table 3:** The co-expression analysis was performed between LYRM4 and the proteins involved in mitochondrial Fe/S protein assembly and export at transcript levels in LIHC using GEPIA2 web

Gene A	Gene B	LIHC tumor		LIHC normal		Liver	
		*p*-value	R	*p*-value	R	*p*-value	R
LYRM4	ISCU	0.011	0.13	9e–08	0.67	0	0.71
	**NFU1**	0	0.45	3.5e–08	0.69	0	0.78
	BOLA1	2.1e–11	0.26	0.00098	0.45	2.8e–06	0.43
	BOLA3	0.00024	0.19	0.29	0.15	4.4e–16	0.68
	NFS1	0.022	0.12	0.32	– 0.14	0.08	0.17
	ACP1	3.5e–09	0.3	0.042	0.29	0	0.7
	ISCA1	0.063	0.097	0.015	0.34	0	0.72
	ISCA2	4.7e–08	0.28	2.4e–05	0.56	2.2e–16	0.68
	IBA57	0.32	0.052	0.34	0.14	0.0011	0.31
	FXN	0.024	0.12	0.19	0.19	0.023	0.22
	FDX1L	2.1e–11	0.34	6.3e–07	0.64	2.1e–14	0.65
	FDXR	0.73	0.018	0.0079	0.37	0.0014	0.3
	HSC20	6.4e–06	0.23	0.016	0.34	8.2e–10	0.54
	HSPA9	0.36	– 0.047	0.3	0.15	4.8e–10	0.55
	GLRX5	0.66	–0.023	0.00061	0.47	2.7e–06	0.43
	ABCB7	0.22	0.064	0.025	0.32	1.1e–09	0.54
	NUBPL	0.13	0.079	0.48	0.1	0.14	0.14
	MFRN1	0.81	0.012	0.027	0.31	0.00035	0.33
	MFRN2	0.48	– 0.037	0.73	0.05	0.31	0.098
	**POLD1**^a^	4.4e–16	0.41	3.6e–07	0.65	0	0.74
	**PRIM2**^a^	0	0.5	0.014	0.35	0	0.74

The bold values indicate the significant difference between the two groups of data, and the statistical significance was set at *p* < 0.05

^a^Represents the key iron-sulphur proteins outside the mitochondria

Cysteine desulfurase activity is crucial for maintaining the iron-sulphur co-factors present in many cell-essential proteins [13, 22]. Furthermore, we found that the mRNA expression of key iron-sulphur proteins NADH dehydrogenase [ubiquinone] iron-sulphur protein 8 (NDUFS8), DNA polymerase delta catalytic subunit (POLD1), and DNA primase large subunit (PRIM2) were significantly elevated (Fig. 5e–g). Moreover, the high mRNA expression of *POLD1* and *PRIM2* was correlated with poor prognosis in LIHC patients (Fig. 5h–i). In addition, we explored the expression patterns of the classical [4Fe-4S] protein ACO2 in the mitochondria of LIHC patients using the Human Protein Atlas. As shown in Fig. 5j, low protein expression of ACO2 was observed in normal hepatocytes, while medium to high protein expression of ACO2 was observed in LIHC tissues. Compared with hepatocytes, the activities of mitochondrial complex I and ACO2 in LIHC cell lines were also significantly elevated (Fig. 5k–l and Additional file 3: Figure S6–13). These results indicated that Fe-S cluster biosynthesis was significantly enhanced in LIHC patients and affected the prognosis of LIHC patients.

### Biological interaction network of LYRM4

**Fig. 6:**
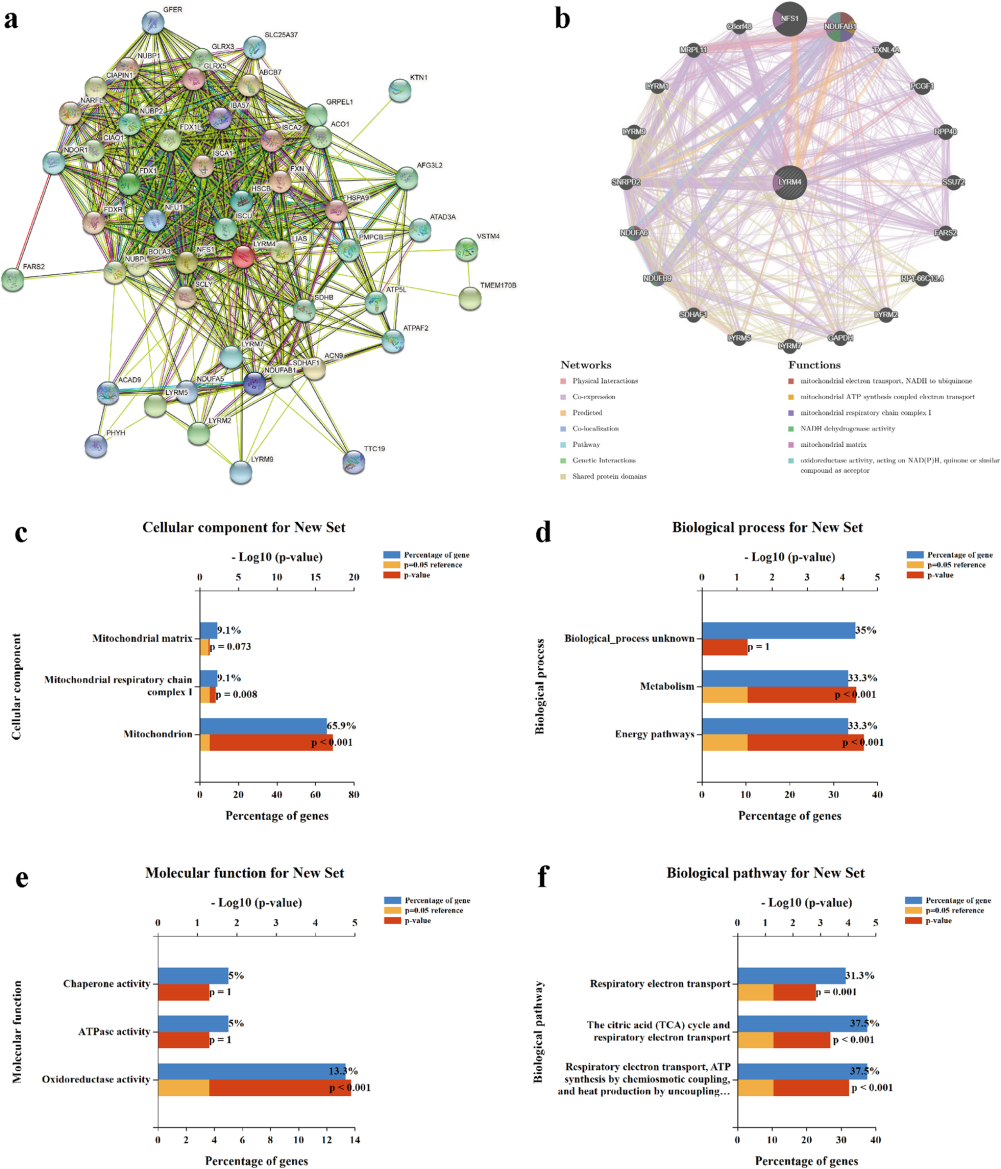
Biological interaction network of LYRM4 and enrichment analysis of gene sets enriched in the target network of LYRM4 (STRING and GeneMANIA databases). **a** Protein network associated with LYRM4. **b** Protein-protein interaction (PPI) network and functional analysis of gene sets enriched in the target network of LYRM4. Colours of the network edges indicate the bioinformatics methods applied: Physical Interactions, Co-expression, Predicted, Co-localization, Pathway, Genetic Interactions, Shared protein domains. Colours of the network nodes indicate the biological functions of enriched gene sets. **c** Biological processes. **d** Cellular components. **e** Molecular functions. **f** KEGG pathway analysis

In order to better understand the molecular function of LYRM4 and the signaling pathways in which LYRM4 is involved, 50 interactors in correlation with LYRM4 were determined using STRING database (Fig. 6a). In addition, protein-protein interaction network analysis via GeneMANIA revealed 20 genes were enriched in LYRM4 network. The enriched gene set plays a role in mitochondrial electron transport, NADH to ubiquinone, mitochondrial ATP synthesis coupled electron transport, mitochondrial respiratory chain complex I and NADH dehydrogenase activity (Fig. 6b). Furthermore, GO enrichment and KEGG pathway analyses for biological interaction network of LYRM4 were performed via FunRich tool. Abundantly enriched GO terms indicated that these genes mainly encode proteins located in the mitochondrion and are involving in energy pathways, metabolism, and oxidoreductase activity (Fig. 6c–e). Similarly, KEGG pathway analysis showed enrichment in respiratory electron transport, ATP synthesis by chemiosmotic coupling, and citric acid cycle signaling pathways (Fig. 6f).

### Enrichment analysis of LYRM4 functional networks in LIHC

#### GO and KEGG pathway analyses of co-expression genes correlated with *LYRM4* in LIHC

**Fig. 7:**
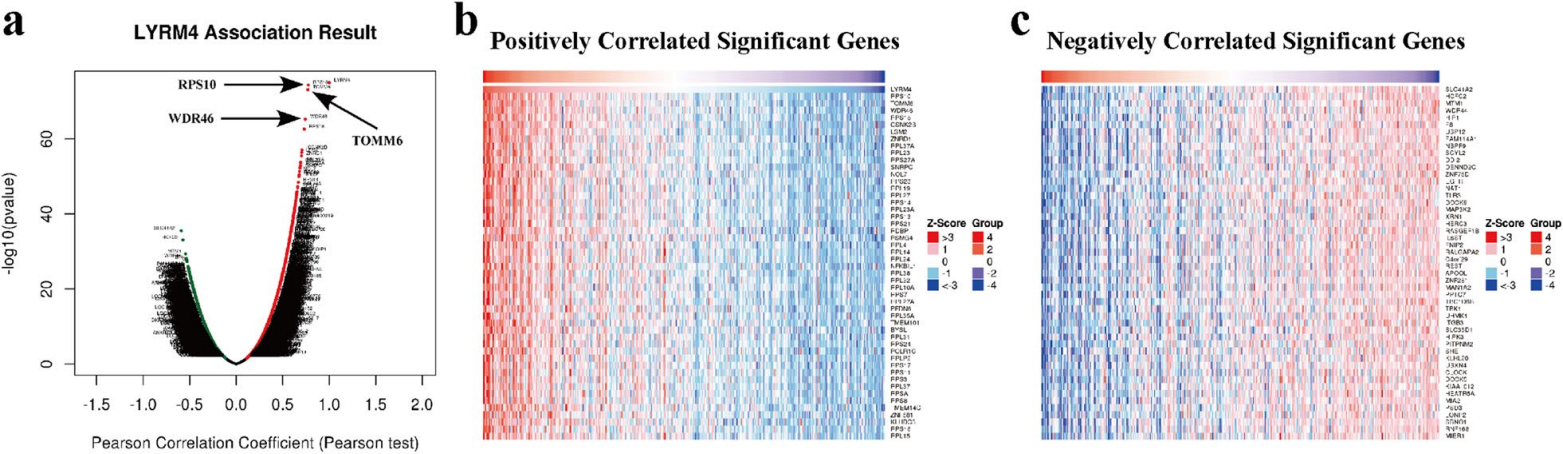
Genes differentially expressed in correlation with *LYRM4* in LIHC (LinkedOmics database). **a** Pearson test was used to analyse correlations between *LYRM4* and genes differentially expressed in LIHC. **b**–**c** Heatmaps showing genes (top 50) that are positively and negatively correlated with *LYRM4* in LIHC. Red indicates positively-correlated genes and blue indicates negatively-correlated genes

**Fig. 8:**
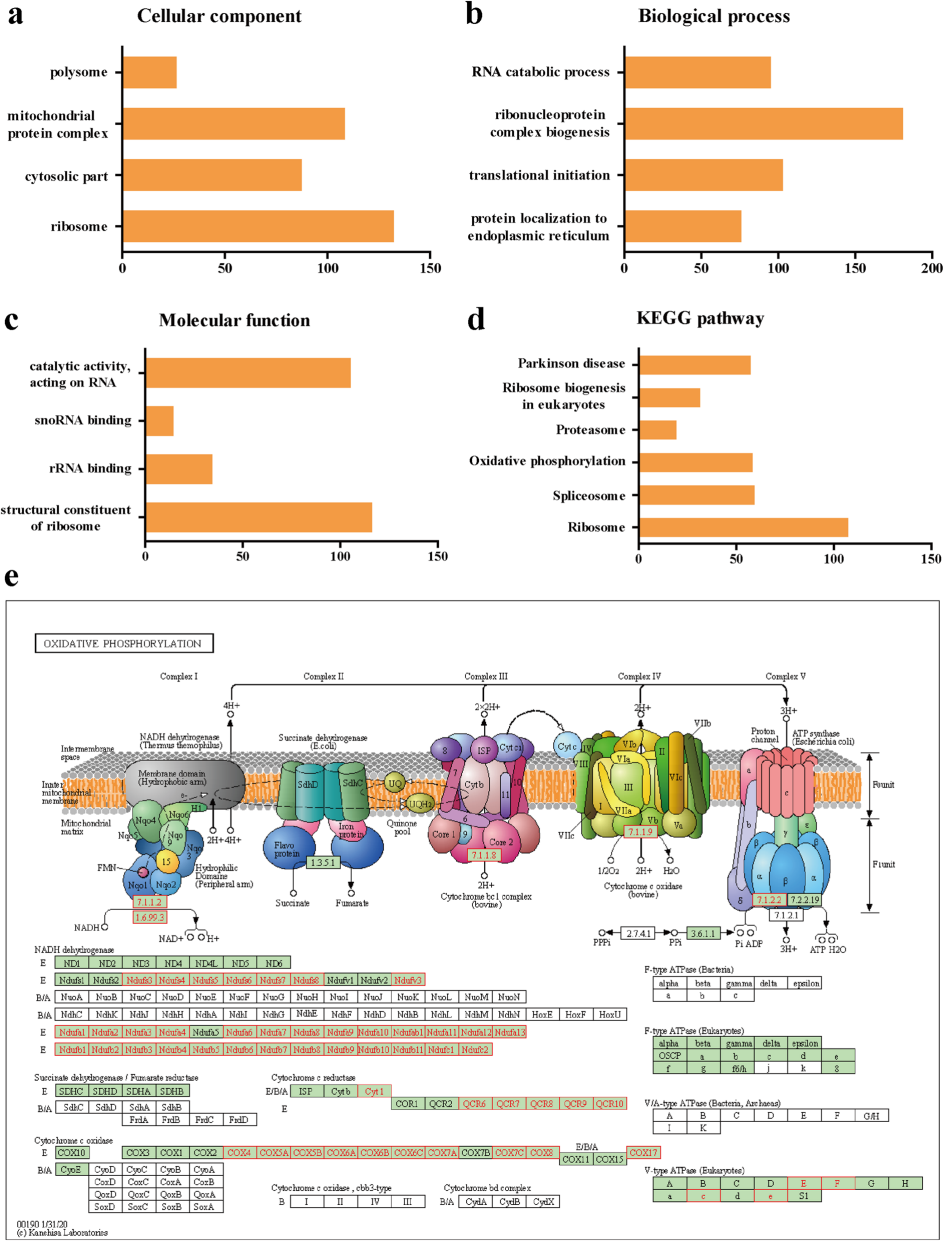
Significantly enriched GO annotations and KEGG pathways of *LYRM4* in LIHC. **a** Cellular components. **b** Biological processes. **c** Molecular functions. **d** KEGG pathway analysis. The orange column represents the Leading Edge Number, and the black represents the false discovery rate (FDR). The FDR from GSEA in the figure is 0. **e** KEGG pathway annotations of the oxidative phosphorylation pathway. The nodes marked in red are associated with the Leading Edge Genes. FDR < 0.05 was considered statistically significant

To analyze the co-expression genes associated with *LYRM4* in LIHC, we used LinkedOmics to analyze mRNA sequencing data from 371 LIHC patients in the TCGA. A Pearson test was conducted to analyse connections among *LYRM4* and genes differentially expressed in LIHC (Fig. 7a). The top 50 significant genes positively or negatively correlated with *LYRM4* were shown in the heatmaps (Fig. 7b-c). These results indicate that the expression of *LYRM4* is closely correlated with a wide range of genes at transcriptome level in LIHC. Three individual genes (RPS10, TOMM6 and WDR46) showed the strongest positive correlation with the expression of *LYRM4* (Additional file 6: Figure S15a–c). This result reflects changes in the component of the 40S ribosomal subunit and mitochondrial protein import, as well as the rRNA modification process in the nucleus and cytosol. Gene Set Enrichment Analysis (GSEA) results demonstrated that differentially expressed genes related to *LYRM4* were mainly located in the ribosomes, cytosolic parts and mitochondrial protein complexes, where they primarily participate in protein localization to endoplasmic reticulum, translational initiation and ribonucleoprotein complex biogenesis. They act as structural constituents of ribosome and rRNA binding (Fig. 8a–c and Additional files 7, 8, 9: Table S3–5). KEGG pathway analysis showed enrichment in the ribosome, spliceosome, oxidative phosphorylation and proteasome pathways (Fig. 8d–e and Additional file 10: Table S6).

### LYRM4 networks of kinase, miRNA or transcription factor targets in LIHC

To further screen the targets of LYRM4 in LIHC, we listed the 5 most significant LYRM4-related genes in the kinase, miRNA and transcription factor target networks in LIHC (Additional files 11, 12, 13: Table S7–9). The results indicated that the most leading edge numbers in kinase target, miRNA target and transcription factor target were Kinase EGFR, AGCACTT, MIR-93, MIR-302A, MIR-302B, MIR-302C, MIR-302D, MIR-372, MIR-373, MIR-520E, MIR-520A, MIR-526B, MIR-520B, MIR-520C, MIR-520D and SCGGAAGY-V$ELK1-02, respectively (Table 4). Since FDR value of the Kinase-target networks of LYRM4 in LIHC was greater than 0.05, we did not carry out subsequent protein-protein interaction (PPI) network construction. The PPI networks constructed by GeneMANIA revealed that gene sets related to miRNA 495 were mainly involved in the regulation of mRNA binding, protein K48-linked deubiquitination, polysome, and maintenance of protein location (Additional file 14: Figure S16). The gene set rich in transcription factor PAX4 is mainly responsible for organ development, mesenchymal cell differentiation and sequence-specific DNA binding (Additional file 15: Figure S17).

**Table 4:** Kinase, miRNA and transcription factor-target networks of LYRM4 in LIHC (LinkedOmics)

Enriched category	Geneset	Leading edge number	FDR	*P* value
Kinase Target	Kinase_MYLK	4	0.11981	0.0045662
	Kinase_MYLK3	4	0.11981	0.0045662
	Kinase_MYLK4	4	0.11981	0.0045662
	Kinase_RPS6KA4	7	0.13792	0.020492
	Kinase_EGFR	26	0.19472	0
miRNA Target	GTTTGTT,MIR-495	97	0	0
	GCACCTT,MIR-18A,MIR-18B	44	0	0
	CTATGCA,MIR-153	92	0	0
	AGCACTT,MIR-93,MIR-302A,MIR-302B,MIR-302C,MIR-302D,MIR-372,MIR-373,MIR-520E,MIR-520A,MIR-526B,MIR-520B,MIR-520C,MIR-520D	160	0	0
	ACATTCC,MIR-1,MIR-206	123	0	0
Transcription Factor Target	GGAANCGGAANY_UNKNOWN	44	0	0
	SCGGAAGY_V$ELK1_02	300	0	0
	V$ISRE_01	78	0.0011582	0
	V$PAX4_02	67	0.0017373	0
	YNGTTNNNATT_UNKNOWN	108	0.0034745	0

FDR, false discovery rate of gene set enrichment analysis (GSEA) from Benjamini and Hochberg. V$, the annotation found in Molecular Signatures Database (MSigDB) for transcription factors (TF)

### miRNAs related to *LYRM4*

**Fig. 9:**
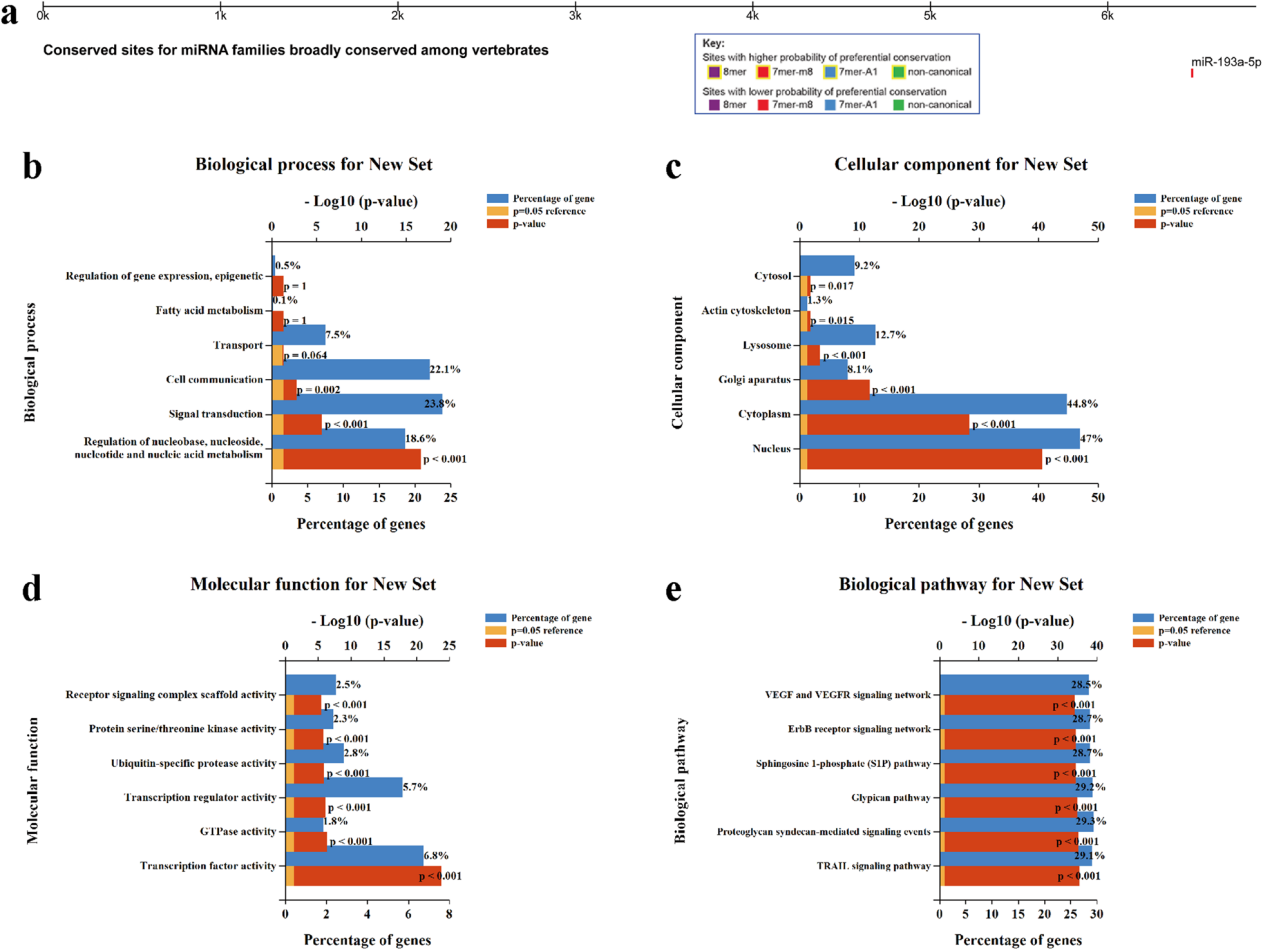
Prediction and enrichment analysis of the identified miRNAs related to *LYRM4* (Targetscan and Funrich). **a** Conserved sites in *LYRM4* for microRNA families broadly conserved among vertebrates. **b**–**e** GO functional and KEGG pathway enrichment analysis of 1,570 identified microRNAs

According to cumulative weighted context++ score, the top 5 among 1570 miRNA families associated with *LYRM4* were miR-24-3p, miR-133a-3p.1, miR-125-5p, miR-193a-5p and miR-302c-3p.2/520-3p. The conserved sites in human *LYRM4* mRNA 3ˊUTR targeted by miRNA families were broadly conserved among vertebrates showed in Fig. 9a. To predict the functional enrichment information of the identified 1570 miRNAs, GO and KEGG pathway analyses were performed using Funrich database. Biological processes were significantly enriched in regulation of nucleobase, nucleoside, nucleotide and nucleic acid metabolism, signal transduction and cell communication. The main enriched Cell components were the nucleus, cytoplasm, and golgi aparatus. In the Molecular function category, transcription factor activity, GTPase activity, and transcription regulator activity were primarily enriched. In terms of KEGG pathway analysis, TRAIL signaling pathway, proteoglycan syndecan-mediated signaling events, glypican pathway and sphingosine 1-phosphate (S1P) pathway were enriched (Fig. 9b–e).

### Immune infiltrates in correlation with *LYRM4* in LIHC

**Fig. 10:**
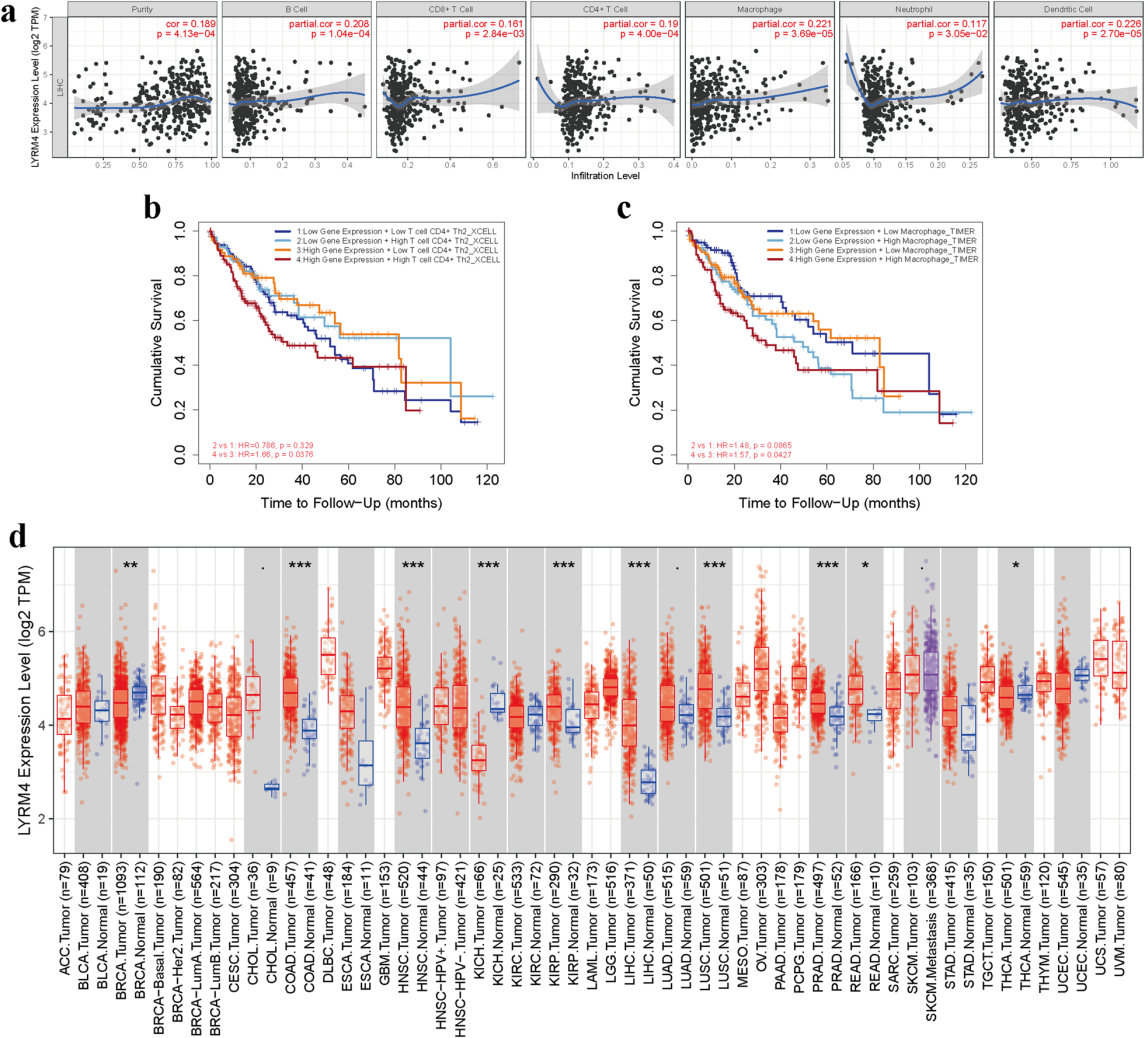
Associations between mRNA expression of *LYRM4* and immune infiltration in LIHC (TIMER&TIMER2.0). **a** Correlation between *LYRM4* expression and the abundance of immune infiltrates (B cells, CD4+ T cells, CD8+ T cells, Neutrophils, Macrophages, and Dendritic cells). Correlation r or purity adjusted r value and *p* value are presented in the figure. The blue line is the fitted curve. **b**–**c** The effect of immune cells infiltration levels in correlation with *LYRM4* expression on the prognosis of LIHC. **d** *LYRM4* mRNA expression in different types of cancers. *, *p* < 0.05; **, *p* < 0.01; ***, *p* < 0.001

LIHC is a common malignant tumour with a poor prognosis. The immune microenvironment is poorly characterised [7]. The expression of *LYRM4* increased significantly with increasing malignancy of LIHC (Fig. 2f–g). Therefore, we explored the relationship between LYRM4 expression and immune infiltration in LIHC. The results showed that the correlation between *LYRM4* expression in LIHC and the infiltration levels of different types of immune cells (B cells, CD4+ T cells, CD8+ T cells, neutrophils, macrophages, and dendritic cells) was statistically significant (*p* < 0.05, Fig. 10a). According to the cumulative survival analysis (Fig. 10b), patients with high LYRM4 expression and high abundance of the T cell CD4+ Th2-XCELL subtype had significantly lower survival rates than those with high LYRM4 expression and low abundance of the T cell CD4+ Th2-XCELL subtype (HR = 1.66, p = 0.0376). The high LYRM4 expression and high abundance of macrophage-TIMER subtype patients exhibited significantly lower survival rates in LIHC patients with high LYRM4 expression and low abundance of macrophage-TIMER subtype (HR = 1.57, p = 0.0427) (Fig. 10c). In addition, the combination of other subtypes and abundance of immune macrophage infiltration with different expression levels of LYRM4 notably affected the survival of LIHC patients (*p* < 0.05, Additional file 16: Figure S18d). Finally, the expression of LYRM4 in pan-cancer was analysed using the TIMER2.0 database. As shown in Fig. 10d, *LYRM4* mRNA was significantly overexpressed in colon adenocarcinoma (COAD), head and neck cancer (HNSC), kidney renal papillary cell carcinoma (KIRP), LIHC, lung squamous cell carcinoma (LUSC), and prostate adenocarcinoma (PRAD; *p* < 0.001).

## Discussion

Differential expression of the proteins required for ISC biosynthesis has been reported to be implications for cellular metabolism and adaptation to cellular stress [22, 42]. Alvarez et al. [43] reported that hypoxia rescues Frataxin loss by restoring ISC biogenesis. In particular, Alvarez et al. [22] found that cancer cells depend on high levels of the ISC biosynthetic enzyme NFS1. Therefore, it is necessary to investigate whether the NFS1-interacting protein LYRM4 is differentially expressed in tumours and its role in carcinogenesis. In this study, we performed bioinformatics analysis using different open databases to acquire detailed information about the potential functions of LYRM4 in LIHC and its regulatory network. We provided the first evidence that the protein expression of LYRM4 is significantly upregulated in LIHC tissues and cell lines (Fig. 5). Thus, this study laid the foundation for future experimental studies on LIHC.

AFP is the most widely used serological indicator for the diagnosis of LIHC worldwide. However, approximately 30%–40% of LIHC patients are AFP-negative, and about 20%–50% of patients with chronic hepatitis or cirrhosis are AFP-positive [44]. A combination of the triple biomarkers AFP, AFP-L3, and DCP was adopted for the early diagnosis of LIHC patients according to the Milan criteria and Child-Pugh class A. The results showed that the 5-year cumulative overall survival (OS) and disease-free survival (DFS) rates (with statistical significance) in postoperative triple-negative and triple-positive patients were 85.3%, 44.2%, 61.7%, and 35.7%, respectively [45]. In addition, the OS of LIHC patients was not significantly different between the high and low AFP expression groups (GEPIA2, data not shown). Therefore, it is necessary to screen for new biomarkers for early LIHC diagnosis and poor patient prognosis.

In this study, we found that *LYRM4* mRNA levels and CNVs were significantly higher in LIHC tissues than in normal tissues (Figs. 1, 2, and Additional file 1: Figure S1). However, overexpression of LYRM4 CNVs was found in only 10% of LIHC samples, based on CNVs (Fig. 1e–f). In particular, the expression level of LYRM4 was positively correlated with the degree of tumour progression and was undifferentiated in LIHC (Fig. 2f–g). We speculate that the overexpression of *LYRM4* in LIHC may be closely related to the significantly reduced promoter DNA methylation levels (Fig. 3b–d).

Interestingly, in the TP53 mutation status subgroup analysis, the transcription level of *LYRM4* was significantly higher in LIHC patients than in healthy people and LIHC patients with no p53 mutation (Fig. 2i). Therefore, these results suggest that TP53 mutant may regulate *LYRM4* expression at the transcriptional level. Funauchi et al. [46] reported that ISCU expression was markedly decreased in the majority of LIHC tissues, and its reduced expression was significantly correlated with p53 mutation. However, in the GEPIA2 database, the mRNA expression level of *ISCU* in LIHC tissues was obviously higher than that in adjacent normal tissues, although the difference was not statistically significant (Additional file 17: Figure S19a). Additionally, analysis of RNA-Seq data from 159 HBV-related LIHC patients showed that *ISCU* mRNA was overexpressed in tumour tissues compared to paired adjacent liver tissues (Additional file 17: Figure S19b), and the Spearman’s correlation coefficient of *the ISCU* mRNA-protein pair was 0.472 (*p* = 8.07E-10) [5]. These results suggest that molecular genetic background, population, whether HBV infection and whether alcoholic fatty liver disease progression of LIHC (Additional file 5: Figure S14) may be the key factors that lead to the abnormal expression of LYRM4 and ISCU in LIHC.

To further investigate the relationship between LYRM4 protein expression and progression of LIHC. We used IHC analysis to indicate that patients with advanced age and higher serum levels of LDL and TG had higher expression of LYRM4 protein (Table 2). We speculated that high expression of LYRM4 may lead to cellular metabolic reprogramming and lipid metabolism disorders by modulating Fe-S cluster assembly, regulating the tricarboxylic acid cycle and β-oxidation [47, 48], which would increase the burden on the liver and thus adversely affect the prognosis of LIHC patients [49, 50]. Unfortunately, we did not find any other significant differences between the clinicopathological and LYRM4 expression. Consistently, Gao et al. [5] reported that Spearman’s correlation coefficient for the *LYRM4* mRNA-protein pair was 0.12832 (*p* = 0.11238) in HBV-related LIHC. These results suggest that the high expression of LYRM4 protein in LIHC may be regulated by post-transcriptional or post-translational modification regulation. It is worthy of further multicentre and large sample size verification.

In conclusion, our data suggest that LYRM4 could serve as a potential diagnostic biomarker for LIHC. Additionally, it may be a useful prognostic marker for LIHC patients, as high expression of *LYRM4* could lead to poor prognosis of LIHC (Fig. 4).

Since ISCs are essential protein cofactors with diverse physiological functions, variations in their abundance may result in changes in various downstream signalling pathways [13]. Clinical mutations of NFS1 (R72Q) [51] and LYRM4 (R68L) [52] could lead to deficits in ISC, thus reducing electron transport chain complex (ETC) activity and mitochondrial respiration. In this study, a biological interaction network of LYRM4-associated genes was constructed, and LYRM4 was involved in the regulation of mitochondrial function and oxidoreductase activity (Fig. 6). These findings are consistent with those of previous reports [10, 52]. It should be noted that the co-expression association between *LYRM4* and 19 genes involved in mitochondrial Fe/S protein assembly and export became gradually disordered with the onset and progression of LIHC (Table 3). Most importantly, we found that the activities of mitochondrial complex I and ACO2 in LIHC were significantly increased. Moreover, the high expression of *POLD1* and *PRIM2* was related to poor clinical outcomes in LIHC (Fig. 5). Therefore, these results indicate that elevated expression of LYRM4 was significantly associated with ISC-specific metabolic reprogramming in LIHC. The regulation of ISC-specific gene set expression was gradually disturbed in paracancerous tissues, which provides a new perspective for preventing the recurrence of LIHC. Meanwhile, we can also select 1-3 iron-sulphur proteins (PRIM2, NFU1, or POLD1), which are highly expressed (Human Protein Atlas) and exhibit a large co-expression correlation coefficient with LYRM4 (Table 3), as a combined biomarkers for the early diagnosis of LIHC with LYRM4, thus effectively improving the specificity and reliability of the diagnostic results.

As shown in Table 4, we found that *LYRM4* in LIHC is linked to a network of miRNAs, including miR-495, miR-18A, miR-18B, and miR-153. Yang et al. [53] found that the upregulation of miR-664, miR-485-3p, and miR-495 leads to a reduced MAT1A expression in LIHC and enhanced tumorigenesis. Xia et al. [54] reported that miR-153 plays a critical role in suppressing epithelial-to-mesenchymal transition (EMT) and LIHC progression by directly downregulating Snail expression. Therefore, we hypothesised that LYRM4 and these miRNAs may have synergistic effects in LIHC. In addition, LYRM4 may downregulate the expression of MAT1A through miR-495, thereby significantly reducing the methylation levels of several proto-oncogene promoter regions. Further studies are needed to confirm this hypothesis.

The EMT signalling pathway plays a critical role in embryological development, cancer progression, and metastasis [55]. A previous study showed that the transcription factor Elk-1 is a downstream target of Erk1/2 MAP kinases, regulating the migration of many types of cancer via its downstream targets including c-fos, EGR1, and PKCα [56]. Zhang et al. [57] demonstrated that upregulation of transcription factor PAX4 directly downregulated miR-144/451 expression and promoted migration and invasion in epithelial cancer cells. It is should also be noted that the mRNA expression of *LYRM4* was significantly associated with tumour thrombus and encapsulation of HBV-related LIHC patients (Table 1). Therefore, we believe that LYRM4 may promote metastasis by regulating the expression of the transcription factors Elk-1 and PAX4 in LIHC (Table 4), which remains to be verified experimentally. Taken together, it is worth exploring whether LYRM4 overexpression mediates malignant transformation and metastasis of LIHC through regulatory metabolic reprogramming and mitochondrial retrograde signalling pathways.

According to reports, the HIF-1α/miR-210 signalling axis regulates mitochondrial free radicals in response to hypoxia in cancer cells by targeting downregulation of ISCU expression, which correlates with poor prognosis in breast cancer and HNSCC [58] and good prognosis in renal cancer [59]. Based on the TargetScan database, miR-193a-5p is the most likely target in miRNA families to regulate *LYRM4* expression (Fig. 9a). Li et al. [60] found that miR-193a-5p was under-expressed in LIHC and inhibited the development of LIHC by targeting SPOCK1. Further functional enrichment of 1570 miRNAs identified as correlated with *LYRM4* revealed terms closely related to the proliferation and apoptosis of LIHC (Fig. 9b–e). Therefore, it will be an interesting project to study the interaction between miR-193a-5p and *LYRM4* in LIHC and its role in the development of LIHC.

Tumour-infiltrating immune cells are highly correlated with prognosis and the identification of immunotherapy targets in LIHC [61]. Our results suggest that immune-infiltrated CD4+ T cells and macrophages substantially affect the prognosis of LIHC and may have a prognostic value related to LYRM4 in LIHC (Fig. 10a–c and Additional file 16: Figure S18). Further studies are needed to verify these findings.

## Conclusions

In summary, we analysed multi-omics data and clinical data from different open databases. IHC was used to explore the correlation between LYRM4 expression and clinical parameters of LIHC patients, demonstrating that LYRM4 plays an important role in hepatocarcinogenesis. In particular, alterations in ISC-specific metabolism in LIHC have been identified. Thus, LYRM4 may be a novel drug target and prognostic biomarker for LIHC. The data acquisition and analysis adopted in this study is time- and cost-efficient, providing a scientific basis for subsequent functional studies. However, functional analysis and biochemical data on the enzymatic activities of ISC biogenesis, tRNA thiolation, and molybdenum cofactor biosynthesis are needed in future studies to confirm the relationship between the overexpression of LYRM4 and the occurrence and development of LIHC in single ethnic groups with or without HBV infection or alcohol abuse.

## Supplementary Information

**Additional file 1: Figure S1.** LYRM4 expression levels in different human cancers and LYRM4 overexpression level in LIHC is negatively correlated with the degree of malignancy of tumor cells.

**Additional file 2: Table S1.** Clinicopathologic Data, related to Table 1.

**Additional file 3: Figure S2–13.** Original data.

**Additional file 4: Table S2.** Clinicopathologic information of immunohistochemical samples, related to Table 2.

**Additional file 5: Figure S14.** Scatterplot of alcohol consumption correlation to LYRM4 protein expression in LIHC patients identified by immunohistochemical staining.

**Additional file 6: Figure S15.** Gene expression correlation analysis for LYRM4, RPS10, TOMM6, and WDR46 (LinkedOmics database).

**Additional file 7: Table S3.** Significantly enriched GO annotations (biological processes) of LYRM4 in LIHC (LinkedOmics).

**Additional file 8: Table S4.** Significantly enriched GO annotations (cellular components) of LYRM4 in LIHC (LinkedOmics).

**Additional file 9: Table S5.** Significantly enriched GO annotations (molecular functions) of LYRM4 in LIHC (LinkedOmics).

**Additional file 10: Table S6.** Significantly enriched KEGG pathway annotations of LYRM4 in LIHC (LinkedOmics).

**Additional file 11: Table S7.** Significantly enriched kinase-target networks of LYRM4 in LIHC (LinkedOmics).

**Additional file 12: Table S8.** Significantly enriched miRNA-target networks of LYRM4 in LIHC (LinkedOmics).

**Additional file 13: Table S9.** Significantly enriched transcription factor-target networks of LYRM4 in LIHC (LinkedOmics).

**Additional file 14: Figure S16.** Protein-protein interaction (PPI) network of miRNA 495-target networks (GeneMANIA database).

**Additional file 15: Figure S17.** PPI network of transcription factor PAX4-target networks (GeneMANIA database).

**Additional file 16: Figure S18.** The effect of LYRM4 expression in correlation with the abundance of immune infiltrates on the prognosis of LIHC (TIMER2.0 database).

**Additional file 17: Figure S19.** IscU mRNA expression levels in LIHC.

## Data Availability

The datasets generated for this study are available on request to the corresponding author.

## Abbreviations

ISC: Iron-sulphur cluster
LIHC: Liver hepatocellular carcinoma
IHC: Immunohistochemical
LDL: Low-density lipoprotein
TG: Triglyceride
HBV: Hepatitis B virus
HCV: Hepatitis C virus
ACP: Acyl carrier protein
FXN: Frataxin
HRP: Horseradish peroxidase
SABC: Streptavidin-biotin-peroxidase complex
DAB: 3,3′-Diaminobenzidine tetrahydrochloride
TMA: Tissue microarrays
DLBC: Lymphoid neoplasm diffuse large B-cell lymphoma
THYM: Thymoma
KICH: Kidney chromophobe
CNV: Copy number variation
PPI: Protein-protein interaction
S1P: Sphingosine 1-phosphate
COAD: Colon adenocarcinoma
HNSC: Head and neck cancer
KIRP: Kidney renal papillary cell carcinoma
LUSC: Lung squamous cell carcinoma
PRAD: Prostate adenocarcinoma
DFS: Disease-free survival
OS: Overall survival
ETC: Electron transport chain complex
EMT: Epithelial-to-mesenchymal transition
FDR: False discovery rate
AFP: Alpha fetoprotein
AFP-L3: *Lens culinaris* agglutinin-reactive fraction of AFP
DCP: Des-γ-carboxy prothrombin
NDUFS8: NADH dehydrogenase [ubiquinone] iron-sulfur protein 8
POLD1: DNA polymerase delta catalytic subunit
PRIM2: DNA primase large subunit
MAT1A: Methionine adenosyltransferase 1A
Elk-1: ETS domain-containing protein
PAX4: Paired box gene 4
