# Supplementary material for: Iron-sulphur cluster biogenesis factor LYRM4 is a novel prognostic biomarker associated with immune infiltrates in hepatocellular carcinoma
Source: Cancer Cell Int. 2021 Sep 6;21:463. doi: 10.1186/s12935-021-02131-3 (PMC8419973; doi:10.1186/s12935-021-02131-3)
Supplement: Supplementary file 1 — Additional file 1: Figure S1. LYRM4 expression levels in different human cancers and LYRM4 overexpression level in LIHC is negatively correlated with the degree of malignancy of tumor cells. [file 12935_2021_2131_MOESM1_ESM.docx]

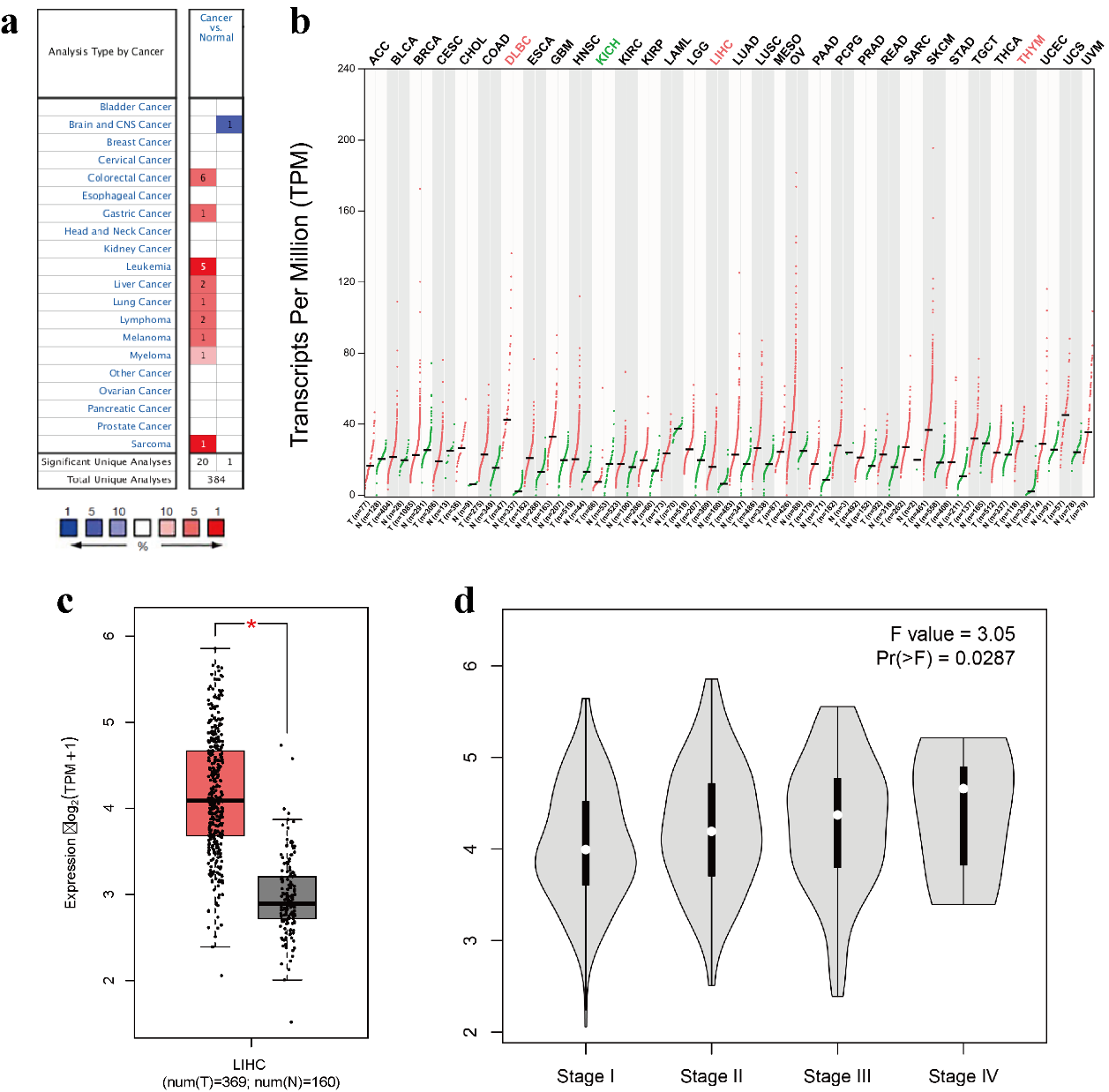


**Additional file 1: Figure S1.** *LYRM4* expression levels in different human cancers and *LYRM4* overexpression level in LIHC is negatively correlated with the degree of malignancy of tumor cells. **a** Comparison of *LYRM4* expression levels in cancer and normal tissue samples based on Oncomine database (Cancer vs normal: the red represents overexpression, the blue represents downexpression). **b** *LYRM4* expression profile across all tumor samples and paired normal tissues determined by GEPIA2 database. **c** Box plot showing *LYRM4* mRNA expression levels in LIHC (GEPIA2). **d** GEPIA2 analysis for the correlation between the expression levels of *LYRM4* mRNA and pathologic staging of LIHC patients. *, *p* < 0.05.
