# Supplementary material for: Iron-sulphur cluster biogenesis factor LYRM4 is a novel prognostic biomarker associated with immune infiltrates in hepatocellular carcinoma
Source: Cancer Cell Int. 2021 Sep 6;21:463. doi: 10.1186/s12935-021-02131-3 (PMC8419973; doi:10.1186/s12935-021-02131-3)
Supplement: Supplementary file 5 — Additional file 5: Figure S14. Scatterplot of alcohol consumption correlation to LYRM4 protein expression in LIHC patients identified by immunohistochemical staining. [file 12935_2021_2131_MOESM5_ESM.docx]

**Additional file 5: Figure S14.** Scatterplot of alcohol consumption correlation to LYRM4 protein expression in LIHC patients identified by immunohistochemical staining. The blue circles represent non-tumor tissues (N), and the red circles represent the paired tumor groups (T). Summary statistics of H-score based on only intact and paired specimens. The number of LIHC patients with a history of alcohol consumption was 32, and the number of non-drinkers was 59. Data are shown as mean ± SD. **, *p* < 0.01; ***, *p* < 0.001.
