# Supplementary material for: Iron-sulphur cluster biogenesis factor LYRM4 is a novel prognostic biomarker associated with immune infiltrates in hepatocellular carcinoma
Source: Cancer Cell Int. 2021 Sep 6;21:463. doi: 10.1186/s12935-021-02131-3 (PMC8419973; doi:10.1186/s12935-021-02131-3)
Supplement: Supplementary file 6 — Additional file 6: Figure S15. Gene expression correlation analysis for LYRM4, RPS10, TOMM6, and WDR46 (LinkedOmics database). [file 12935_2021_2131_MOESM6_ESM.docx]

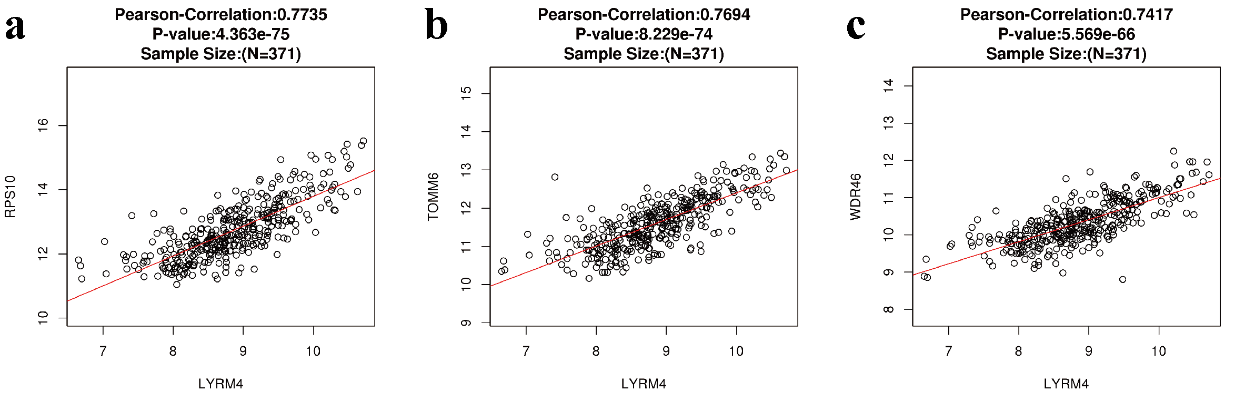


**Additional file 6: Figure S15.** Gene expression correlation analysis for *LYRM4*, RPS10, TOMM6, and WDR46 (LinkedOmics database). The scatter plots show that *LYRM4* expression is positively correlated with the expression of RPS10 **(a)**, TOMM6 **(b)**, and WDR46 **(c)**.
