# Supplementary material for: Iron-sulphur cluster biogenesis factor LYRM4 is a novel prognostic biomarker associated with immune infiltrates in hepatocellular carcinoma
Source: Cancer Cell Int. 2021 Sep 6;21:463. doi: 10.1186/s12935-021-02131-3 (PMC8419973; doi:10.1186/s12935-021-02131-3)
Supplement: Supplementary file 7 — Additional file 7: Table S3. Significantly enriched GO annotations (biological processes) of LYRM4 in LIHC (LinkedOmics). [file 12935_2021_2131_MOESM7_ESM.docx]

**Additional file 7: Table S3.** Significantly enriched GO annotations (biological processes) of *LYRM4* in LIHC (LinkedOmics)

| **Description** | **Leading Edge**  **Number** | **FDR** | **Leading Edge Gene** |
| --- | --- | --- | --- |
| protein localization to endoplasmic reticulum | 77 | 0 | KDELR1; RPL10; RPL10A; RPL11; RPL12; RPL13; RPL13A; RPL14; RPL15; RPL17; RPL18; RPL18A; RPL19; RPL21; RPL22; RPL23; RPL23A; RPL24; RPL26; RPL27; RPL27A; RPL28; RPL29; RPL3; RPL30; RPL31; RPL32; RPL34; RPL35; RPL35A; RPL36; RPL37; RPL37A; RPL38; RPL39; RPL4; RPL41; RPL5; RPL6; RPL7; RPL7A; RPL8; RPLP0; RPLP1; RPLP2; RPS10; RPS11; RPS12; RPS13; RPS14; RPS15; RPS15A; RPS16; RPS17; RPS18; RPS19; RPS2; RPS20; RPS21; RPS23; RPS24; RPS25; RPS27A; RPS29; RPS3; RPS3A; RPS4X; RPS5; RPS6; RPS7; RPS8; RPS9; RPSA; SEC61G; SRP14; UBA52; ZFAND2B |
| translational initiation | 104 | 0 | ATF4; CDC123; EIF1; EIF1AD; EIF2B4; EIF2B5; EIF2S2; EIF3B; EIF3D; EIF3E; EIF3F; EIF3G; EIF3H; EIF3K; EIF3L; EIF3M; EIF4A1; EIF4B; EIF4E2; EIF4EBP3; EIF5B; EIF6; HSPB1; NPM1; PABPC1; POLR2D; POLR2G; PPP1CA; RBM4; RPL10; RPL10A; RPL11; RPL12; RPL13; RPL13A; RPL14; RPL15; RPL17; RPL18; RPL18A; RPL19; RPL21; RPL22; RPL23; RPL23A; RPL24; RPL26; RPL27; RPL27A; RPL28; RPL29; RPL3; RPL30; RPL31; RPL32; RPL34; RPL35; RPL35A; RPL36; RPL37; RPL37A; RPL38; RPL39; RPL4; RPL41; RPL5; RPL6; RPL7; RPL7A; RPL8; RPLP0; RPLP1; RPLP2; RPS10; RPS11; RPS12; RPS13; RPS14; RPS15; RPS15A; RPS16; RPS17; RPS18; RPS19; RPS2; RPS20; RPS21; RPS23; RPS24; RPS25; RPS26; RPS27; RPS27A; RPS29; RPS3; RPS3A; RPS4X; RPS5; RPS6; RPS7; RPS8; RPS9; RPSA; UBA52 |
| ribonucleoprotein complex biogenesis | 182 | 0 | AAMP; AATF; ABT1; BCCIP; BMS1; BRIX1; BYSL; C1QBP; CD2BP2; CIRBP; CLNS1A; CRNKL1; DCAF13; DDX27; DDX49; DDX51; DDX56; DIS3L2; EDC3; EIF2S3; EIF3B; EIF3CL; EIF3D; EIF3E; EIF3F; EIF3G; EIF3H; EIF3K; EIF3L; EIF3M; EIF4A3; EIF4B; EIF6; EMG1; ERAL1; ERI3; EXOSC1; EXOSC2; EXOSC4; EXOSC5; EXOSC6; EXOSC7; EXOSC8; FBL; FRG1; FTSJ3; GAR1; GEMIN6; GEMIN7; GTF3A; HSP90AB1; IMP3; IMP4; KRI1; LSM3; LSM4; LYAR; MRM1; MRPL1; MRPL10; MRPL11; MRPL20; MRPL22; MRPS11; MRPS2; MRPS7; MRPS9; MRTO4; NAT10; NGDN; NHP2; NLE1; NOB1; NOC4L; NOL11; NOL12; NOP10; NOP16; NOP2; NOP56; NOP58; NPM1; NPM3; NSA2; NSUN5; PA2G4; PAK1IP1; PDCD11; PELP1; PES1; PIH1D1; POLR2D; POP4; POP5; PPAN; PRPF19; PRPF31; PRPF6; PWP1; RAN; RBM22; REXO4; RIOK1; RPF2; RPL10; RPL10A; RPL11; RPL12; RPL13A; RPL14; RPL23A; RPL24; RPL26; RPL26L1; RPL27; RPL3; RPL35; RPL35A; RPL38; RPL5; RPL6; RPL7; RPL7A; RPL7L1; RPLP0; RPS10; RPS14; RPS15; RPS16; RPS17; RPS19; RPS2; RPS21; RPS23; RPS24; RPS27; RPS5; RPS6; RPS7; RPS8; RPS9; RPSA; RPUSD2; RRP1; RRP7A; RRP8; RRP9; RRS1; RSL1D1; RSL24D1; RUVBL1; RUVBL2; SART1; SF3A2; SNRPB; SNRPC; SNRPD1; SNRPD2; SNRPD3; SNRPE; SNRPF; SNRPG; SNUPN; SURF6; SUV39H1; TAF9; TARBP2; TBL3; TRMT112; TXNL4A; UTP18; UTP6; WDR12; WDR18; WDR46; WDR74; WDR75; XAB2; ZNF593; ZNF622; ZNHIT3; ZRSR2 |
| RNA catabolic process | 96 | 0 | ANP32A; EDC3; EIF3E; EXOSC1; EXOSC4; EXOSC5; EXOSC7; EXOSC8; HSPB1; LSM2; LSM3; LSM4; LSM5; LSM7; MRTO4; NANOS1; NPM1; PCBP4; POLR2G; PPP2R1A; RNASEH2C; RPL10; RPL10A; RPL11; RPL12; RPL13; RPL13A; RPL14; RPL15; RPL17; RPL18; RPL18A; RPL19; RPL21; RPL22; RPL23; RPL23A; RPL24; RPL26; RPL27; RPL27A; RPL28; RPL29; RPL3; RPL30; RPL31; RPL32; RPL34; RPL35; RPL35A; RPL36; RPL37; RPL37A; RPL38; RPL39; RPL4; RPL41; RPL5; RPL6; RPL7; RPL7A; RPL8; RPLP0; RPLP1; RPLP2; RPS10; RPS11; RPS12; RPS13; RPS14; RPS15; RPS15A; RPS16; RPS17; RPS18; RPS19; RPS2; RPS20; RPS21; RPS23; RPS24; RPS25; RPS27A; RPS29; RPS3; RPS3A; RPS4X; RPS5; RPS6; RPS7; RPS8; RPS9; RPSA; SKIV2L; TBRG4; UBA52 |

Abbreviations: FDR, False discovery rate of Gene Set Enrichment Analysis (GSEA) from Benjamini and Hochberg.
