# Supplementary material for: Iron-sulphur cluster biogenesis factor LYRM4 is a novel prognostic biomarker associated with immune infiltrates in hepatocellular carcinoma
Source: Cancer Cell Int. 2021 Sep 6;21:463. doi: 10.1186/s12935-021-02131-3 (PMC8419973; doi:10.1186/s12935-021-02131-3)
Supplement: Supplementary file 8 — Additional file 8: Table S4. Significantly enriched GO annotations (cellular components) of LYRM4 in LIHC (LinkedOmics). [file 12935_2021_2131_MOESM8_ESM.docx]

**Additional file 8: Table S4.** Significantly enriched GO annotations (cellular components) of *LYRM4* in LIHC (LinkedOmics).

| **Description** | **Leading Edge**  **Number** | **FDR** | **Leading Edge Gene** |
| --- | --- | --- | --- |
| ribosome | 133 | 0 | ABCF1; APEX1; CHCHD1; EIF3H; GADD45GIP1; MRPL10; MRPL11; MRPL12; MRPL13; MRPL14; MRPL17; MRPL2; MRPL21; MRPL22; MRPL23; MRPL24; MRPL27; MRPL28; MRPL30; MRPL33; MRPL36; MRPL38; MRPL40; MRPL43; MRPL47; MRPL48; MRPL51; MRPL52; MRPL53; MRPL54; MRPL55; MRPL9; MRPS10; MRPS11; MRPS12; MRPS15; MRPS16; MRPS17; MRPS18A; MRPS18B; MRPS21; MRPS23; MRPS24; MRPS25; MRPS26; MRPS33; MRPS34; MRPS5; MRPS7; MRPS9; MTG1; NDUFA7; RPL10; RPL10A; RPL11; RPL12; RPL13; RPL13A; RPL14; RPL15; RPL17; RPL18; RPL18A; RPL19; RPL21; RPL22; RPL22L1; RPL23; RPL23A; RPL24; RPL26; RPL26L1; RPL27; RPL27A; RPL28; RPL29; RPL3; RPL30; RPL31; RPL32; RPL34; RPL35; RPL35A; RPL36; RPL36AL; RPL37; RPL37A; RPL38; RPL39; RPL4; RPL41; RPL5; RPL6; RPL7; RPL7A; RPL8; RPLP0; RPLP1; RPLP2; RPS10; RPS11; RPS12; RPS13; RPS14; RPS15; RPS15A; RPS16; RPS17; RPS18; RPS19; RPS2; RPS20; RPS21; RPS23; RPS24; RPS25; RPS26; RPS27; RPS27A; RPS29; RPS3; RPS3A; RPS4X; RPS5; RPS6; RPS7; RPS8; RPS9; RPSA; RSL24D1; SURF6; UBA52; ZNF622 |
| cytosolic part | 88 | 0 | BLOC1S3; CCT3; CCT4; CCT7; CTU1; DTNBP1; PIN1; PSMC5; RPL10; RPL10A; RPL11; RPL12; RPL13; RPL13A; RPL14; RPL15; RPL17; RPL18; RPL18A; RPL19; RPL21; RPL22; RPL22L1; RPL23; RPL23A; RPL24; RPL26; RPL26L1; RPL27; RPL27A; RPL28; RPL29; RPL3; RPL30; RPL31; RPL32; RPL34; RPL35; RPL35A; RPL36; RPL36AL; RPL37; RPL37A; RPL38; RPL39; RPL4; RPL41; RPL5; RPL6; RPL7; RPL7A; RPL8; RPLP0; RPLP1; RPLP2; RPS10; RPS11; RPS12; RPS13; RPS14; RPS15; RPS15A; RPS16; RPS17; RPS18; RPS19; RPS2; RPS20; RPS21; RPS23; RPS24; RPS25; RPS27A; RPS29; RPS3; RPS3A; RPS4X; RPS5; RPS6; RPS7; RPS8; RPS9; RPSA; RSL24D1; SURF6; UBA52; UBQLN4; ZNF622 |
| mitochondrial protein complex | 109 | 0 | ANKZF1; BAX; BCS1L; CHCHD1; CHCHD6; COX4I1; COX4I2; COX5A; COX5B; COX7A2L; COX7C; CYC1; IMMP1L; MRPL10; MRPL11; MRPL12; MRPL13; MRPL14; MRPL17; MRPL2; MRPL20; MRPL21; MRPL22; MRPL23; MRPL24; MRPL27; MRPL28; MRPL30; MRPL33; MRPL36; MRPL38; MRPL40; MRPL43; MRPL47; MRPL48; MRPL51; MRPL52; MRPL53; MRPL54; MRPL55; MRPL9; MRPS10; MRPS11; MRPS12; MRPS15; MRPS16; MRPS17; MRPS18A; MRPS18B; MRPS18C; MRPS2; MRPS21; MRPS24; MRPS26; MRPS27; MRPS33; MRPS34; MRPS5; MRPS7; MRPS9; MTG1; MTX1; NDUFA11; NDUFA13; NDUFA2; NDUFA3; NDUFA6; NDUFA7; NDUFA8; NDUFB1; NDUFB10; NDUFB11; NDUFB2; NDUFB3; NDUFB4; NDUFB5; NDUFB7; NDUFB8; NDUFB9; NDUFC1; NDUFC2; NDUFS3; NDUFS4; NDUFS5; NDUFS6; NDUFS8; NDUFV3; POLRMT; ROMO1; SLC25A6; SUPV3L1; TIMM10; TIMM13; TIMM17B; TIMM22; TIMM50; TIMM8B; TIMM9; TOMM20; TOMM20L; TOMM22; TOMM40; TOMM5; TOMM6; TOMM7; UQCR10; UQCRB; UQCRH; UQCRHL |
| polysome | 27 | 0 | DRG1; EIF3H; MCRS1; NAA38; RPL10A; RPL11; RPL18; RPL18A; RPL19; RPL24; RPL30; RPL31; RPL32; RPL36; RPL38; RPL39; RPL41; RPL6; RPL7; RPL7A; RPL8; RPS21; RPS23; RPS29; RPS3; RPS4X; RPS6 |
