# Supplementary material for: Iron-sulphur cluster biogenesis factor LYRM4 is a novel prognostic biomarker associated with immune infiltrates in hepatocellular carcinoma
Source: Cancer Cell Int. 2021 Sep 6;21:463. doi: 10.1186/s12935-021-02131-3 (PMC8419973; doi:10.1186/s12935-021-02131-3)
Supplement: Supplementary file 9 — Additional file 9: Table S5. Significantly enriched GO annotations (molecular functions) of LYRM4 in LIHC (LinkedOmics). [file 12935_2021_2131_MOESM9_ESM.docx]

**Additional file 9: Table S5.** Significantly enriched GO annotations (molecular functions) of *LYRM4* in LIHC (LinkedOmics).

| **Description** | **Leading Edge**  **Number** | **FDR** | **Leading Edge Gene** |
| --- | --- | --- | --- |
| structural constituent of ribosome | 117 | 0 | MRPL10; MRPL11; MRPL12; MRPL13; MRPL14; MRPL17; MRPL2; MRPL21; MRPL22; MRPL23; MRPL24; MRPL27; MRPL28; MRPL30; MRPL33; MRPL36; MRPL43; MRPL47; MRPL51; MRPL52; MRPL55; MRPL9; MRPS11; MRPS12; MRPS15; MRPS16; MRPS17; MRPS18A; MRPS18B; MRPS21; MRPS23; MRPS24; MRPS25; MRPS33; MRPS34; MRPS5; MRPS7; MRPS9; NDUFA7; RPL10; RPL10A; RPL11; RPL12; RPL13; RPL13A; RPL14; RPL15; RPL17; RPL18; RPL18A; RPL19; RPL21; RPL22; RPL22L1; RPL23; RPL23A; RPL24; RPL26; RPL26L1; RPL27; RPL27A; RPL28; RPL29; RPL3; RPL30; RPL31; RPL32; RPL34; RPL35; RPL35A; RPL36; RPL36AL; RPL37; RPL37A; RPL38; RPL39; RPL4; RPL41; RPL5; RPL6; RPL7; RPL7A; RPL8; RPLP0; RPLP1; RPLP2; RPS10; RPS11; RPS12; RPS13; RPS14; RPS15; RPS15A; RPS16; RPS17; RPS18; RPS19; RPS2; RPS20; RPS21; RPS23; RPS24; RPS26; RPS27; RPS27A; RPS29; RPS3; RPS3A; RPS4X; RPS5; RPS6; RPS7; RPS8; RPS9; RPSA; RSL24D1; UBA52 |
| rRNA binding | 35 | 0 | EMG1; ERAL1; GTF3A; IMP3; IMP4; MRPL11; MRPL20; MRPS11; MRPS17; MRPS18A; MRPS18C; MRPS27; MRPS7; NGRN; NOL12; PPAN; RPF2; RPL11; RPL12; RPL19; RPL23; RPL23A; RPL37; RPL5; RPL8; RPLP0; RPS11; RPS13; RPS14; RPS18; RPS3; RPS4X; RPS5; RPS9; RRS1 |
| snoRNA binding | 15 | 0 | BMS1; BYSL; GAR1; IMP3; IMP4; NHP2; NOP10; NOP56; NOP58; NUDT1; NUDT16L1; NUDT5; RRP9; TBL3; UTP6 |
| catalytic activity, acting on RNA | 106 | 0 | AARS2; APEX1; CARS2; CDKAL1; CPSF3; DALRD3; DDX10; DDX54; DDX56; DHX16; DHX30; DHX34; DHX37; DIS3L2; DQX1; DTD1; DUS1L; DUS3L; EDC3; EIF4A1; EIF4A3; EMG1; ENDOG; ERI3; EXOSC1; EXOSC2; EXOSC3; EXOSC4; EXOSC5; EXOSC6; EXOSC7; EXOSC8; EXOSC9; FARS2; FARSA; FARSB; FBL; FTSJ3; GARS; HARS; KARS; MED20; MEPCE; METTL1; METTL2A; METTL6; MRM1; NOB1; NOP2; POLR1C; POLR1D; POLR1E; POLR2D; POLR2E; POLR2F; POLR2G; POLR2H; POLR2I; POLR2J; POLR2K; POLR2L; POLR3C; POLR3F; POLR3H; POLRMT; POP4; POP5; POP7; PRIM2; PTRH1; PTRH2; PUS1; QARS; QTRT1; RARS; RBMX2; RNASEH1; RNASEH2A; RNASEH2B; RNASEK; RNASET2; RPP21; RPP30; RPP38; RPP40; SARS2; SKIV2L; SND1; SUPV3L1; TDP2; TERT; THUMPD2; TRMT1; TRMT112; TRMT12; TRMT2A; TRMT61A; TRPT1; TSEN2; TSEN34; TSEN54; TSN; VARS; VARS2; WDR4; ZNRD1 |
