# Supplementary material for: Iron-sulphur cluster biogenesis factor LYRM4 is a novel prognostic biomarker associated with immune infiltrates in hepatocellular carcinoma
Source: Cancer Cell Int. 2021 Sep 6;21:463. doi: 10.1186/s12935-021-02131-3 (PMC8419973; doi:10.1186/s12935-021-02131-3)
Supplement: Supplementary file 10 — Additional file 10: Table S6. Significantly enriched KEGG pathway annotations of LYRM4 in LIHC (LinkedOmics). [file 12935_2021_2131_MOESM10_ESM.docx]

**Additional file 10: Table S6.** Significantly enriched KEGG pathway annotations of *LYRM4* in LIHC (LinkedOmics).

| **Description** | **Leading Edge**  **Number** | **FDR** | **Leading Edge Gene** |
| --- | --- | --- | --- |
| Ribosome | 108 | 0 | FAU; MRPL10; MRPL11; MRPL12; MRPL13; MRPL14; MRPL17; MRPL2; MRPL21; MRPL22; MRPL23; MRPL24; MRPL27; MRPL28; MRPL30; MRPL33; MRPL36; MRPL9; MRPS10; MRPS11; MRPS12; MRPS15; MRPS16; MRPS17; MRPS18A; MRPS21; MRPS5; MRPS7; MRPS9; RPL10; RPL10A; RPL11; RPL12; RPL13; RPL13A; RPL14; RPL15; RPL17; RPL18; RPL18A; RPL19; RPL21; RPL22; RPL22L1; RPL23; RPL23A; RPL24; RPL26; RPL26L1; RPL27; RPL27A; RPL28; RPL29; RPL3; RPL30; RPL31; RPL32; RPL34; RPL35; RPL35A; RPL36; RPL36AL; RPL37; RPL37A; RPL38; RPL39; RPL4; RPL41; RPL5; RPL6; RPL7; RPL7A; RPL8; RPLP0; RPLP1; RPLP2; RPS10; RPS11; RPS12; RPS13; RPS14; RPS15; RPS15A; RPS16; RPS17; RPS18; RPS19; RPS2; RPS20; RPS21; RPS23; RPS24; RPS25; RPS26; RPS27; RPS27A; RPS29; RPS3; RPS3A; RPS4X; RPS5; RPS6; RPS7; RPS8; RPS9; RPSA; RSL24D1; UBA52 |
| Spliceosome | 60 | 0 | BUD31; CCDC12; CRNKL1; CTNNBL1; CWC15; DHX16; EFTUD2; EIF4A3; HNRNPA1; HNRNPA1L2; HNRNPA3; HNRNPC; HSPA1B; HSPA1L; ISY1; LSM2; LSM3; LSM4; LSM5; LSM6; LSM7; MAGOH; MAGOHB; NCBP2; PHF5A; PPIE; PPIH; PPIL1; PQBP1; PRPF19; PRPF31; PRPF6; PUF60; RBM22; RBM8A; RBMX; RP9; SART1; SF3A2; SF3B4; SF3B5; SNRNP70; SNRPA; SNRPA1; SNRPB; SNRPB2; SNRPC; SNRPD1; SNRPD2; SNRPD3; SNRPE; SNRPF; SNRPG; SYF2; THOC3; TXNL4A; U2AF1; U2AF1L4; XAB2; ZMAT2 |
| Oxidative phosphorylation | 59 | 0 | ATP6V0B; ATP6V0C; ATP6V0E1; ATP6V1E1; ATP6V1F; COX17; COX4I1; COX4I2; COX5A; COX5B; COX6A1; COX6B1; COX6C; COX7A1; COX7A2; COX7A2L; COX7C; COX8A; CYC1; NDUFA1; NDUFA10; NDUFA11; NDUFA12; NDUFA13; NDUFA2; NDUFA3; NDUFA4; NDUFA4L2; NDUFA6; NDUFA7; NDUFA8; NDUFA9; NDUFAB1; NDUFB1; NDUFB10; NDUFB11; NDUFB2; NDUFB3; NDUFB4; NDUFB5; NDUFB6; NDUFB7; NDUFB8; NDUFB9; NDUFC1; NDUFC2; NDUFS3; NDUFS4; NDUFS5; NDUFS6; NDUFS7; NDUFS8; NDUFV3; UQCR10; UQCR11; UQCRB; UQCRH; UQCRHL; UQCRQ |
| Proteasome | 20 | 0 | ADRM1; POMP; PSMA4; PSMA6; PSMA7; PSMB1; PSMB3; PSMB4; PSMB5; PSMB6; PSMB7; PSMC1; PSMC3; PSMC4; PSMC5; PSMD13; PSMD4; PSMD8; PSME2; PSMF1 |
| Ribosome biogenesis in eukaryotes | 32 | 0 | BMS1; CSNK2A2; CSNK2B; EIF6; EMG1; FBL; GAR1; GNL3; IMP3; IMP4; NAT10; NHP2; NOB1; NOP10; NOP56; NOP58; NXT1; POP4; POP5; POP7; RAN; RIOK1; RMRP; RPP30; RPP38; RPP40; RRP7A; TBL3; TCOF1; UTP18; UTP6; WDR75 |
| Parkinson disease | 58 | 0 | COX4I1; COX4I2; COX5A; COX5B; COX6A1; COX6B1; COX6C; COX7A1; COX7A2; COX7A2L; COX7C; COX8A; CYC1; HTRA2; NDUFA1; NDUFA10; NDUFA11; NDUFA12; NDUFA13; NDUFA2; NDUFA3; NDUFA4; NDUFA4L2; NDUFA6; NDUFA7; NDUFA8; NDUFA9; NDUFAB1; NDUFB1; NDUFB10; NDUFB11; NDUFB2; NDUFB3; NDUFB4; NDUFB5; NDUFB6; NDUFB7; NDUFB8; NDUFB9; NDUFC1; NDUFC2; NDUFS3; NDUFS4; NDUFS5; NDUFS6; NDUFS7; NDUFS8; NDUFV3; SLC25A6; UBE2J2; UBE2L3; UQCR10; UQCR11; UQCRB; UQCRH; UQCRHL; UQCRQ; VDAC2 |
