# Supplementary material for: Iron-sulphur cluster biogenesis factor LYRM4 is a novel prognostic biomarker associated with immune infiltrates in hepatocellular carcinoma
Source: Cancer Cell Int. 2021 Sep 6;21:463. doi: 10.1186/s12935-021-02131-3 (PMC8419973; doi:10.1186/s12935-021-02131-3)
Supplement: Supplementary file 11 — Additional file 11: Table S7. Significantly enriched kinase-target networks of LYRM4 in LIHC (LinkedOmics). [file 12935_2021_2131_MOESM11_ESM.docx]

**Additional file 11: Table S7.** Significantly enriched kinase-target networks of *LYRM4* in LIHC (LinkedOmics).

| **Geneset** | **Leading Edge Gene** |
| --- | --- |
| Kinase_MYLK | MYL12B; MYL5; MYL6; MYL6B |
| Kinase_MYLK3 | MYL12B; MYL5; MYL6; MYL6B |
| Kinase_MYLK4 | MYL12B; MYL5; MYL6; MYL6B |
| Kinase_RPS6KA4 | ATF4; H3F3A; H3F3B; HIST1H3C; HIST1H3F; HIST1H3I; HMGN1 |
| Kinase_EGFR | ABCA1; ANXA1; ASAP3; ATM; CBL; CBLB; CCDC50; CRK; CRKL; CTNNB1; CTNND1; EGFR; EPB41; EZR; GAB1; HDAC6; KCND3; KCNJ4; LRRK1; LYN; MAP2K1; PLCG2; PTPN1; SHC4; STAT5B; TLR3 |
