# Supplementary material for: Iron-sulphur cluster biogenesis factor LYRM4 is a novel prognostic biomarker associated with immune infiltrates in hepatocellular carcinoma
Source: Cancer Cell Int. 2021 Sep 6;21:463. doi: 10.1186/s12935-021-02131-3 (PMC8419973; doi:10.1186/s12935-021-02131-3)
Supplement: Supplementary file 12 — Additional file 12: Table S8. Significantly enriched miRNA-target networks of LYRM4 in LIHC (LinkedOmics). [file 12935_2021_2131_MOESM12_ESM.docx]

**Additional file 12: Table S8.** Significantly enriched miRNA-target networks of *LYRM4* in LIHC (LinkedOmics).

| **Geneset** | **Leading Edge Gene** |
| --- | --- |
| GTTTGTT,MIR-495 | ABCA2; ACVR1B; ANKRD13C; AP1G1; APPBP2; ARHGAP5; ARMC8; ATP2B2; ATRN; C18orf25; CADM1; CAPZA1; CBL; CD164; CELF1; CPEB3; CREBZF; DAG1; DDX3X; DNAJC14; DUSP6; DYRK1A; EPC2; ETS1; FAM122B; FAM126B; FKBP5; GLCCI1; GPM6A; HIPK1; HMGXB4; HNRNPH2; HSDL2; IGF1; IREB2; KCNJ2; KLF13; LHX2; MAML3; MAPK6; MBNL2; MDM1; MGAT2; MORF4L2; MTSS1; MYLK; NAA30; NCOA1; NFAT5; NHLRC3; NRBF2; NUFIP2; PCDHAC1; PCDHAC2; PHF6; PNN; R3HDM2; RAI2; RBPMS2; REV1; RICTOR; RLF; RND3; RORB; RSBN1; RUNX1T1; S1PR3; SATB1; SCN8A; SEL1L; SEMA6D; SEPT7; SGK1; SGMS1; SLC38A2; SOX5; SOX6; SP1; SP4; SSX2IP; STAG2; STRN; THBS2; TNRC6A; TRAPPC10; TSC22D3; TSHZ3; TWF1; UBE3A; UBN1; USP32; VCPIP1; VPS13D; WDTC1; YTHDC1; ZFAND5; ZFX |
| GCACCTT,MIR-18A,MIR-18B | AKR1D1; ANKRD50; ARL15; ASXL2; BHLHE22; BRWD3; CLASP2; CREBL2; DIP2C; EHMT1; ESR1; FCHSD2; GAB1; HIF1A; HMBOX1; IGF1; IRF2; KDM2A; KLHL20; MAN1A2; NAV1; NCOA1; NFAT5; NR3C1; PDZD2; PHF2; PHF20L1; PRKACB; PSD3; RAB11FIP2; SH3BP4; SMAP2; SOCS5; SON; SORBS2; STK4; TEX2; TNRC6B; TRIM2; TSHZ3; ZBTB4; ZBTB44; ZFP36L1; ZNF704 |
| CTATGCA,MIR-153 | ADAM19; AKAP6; APC; APLP2; APP; ARHGAP5; ARHGAP6; AUTS2; BMPR2; BPTF; BTBD7; C6orf120; CDK13; CELF2; CITED2; CLTC; CMIP; CNN3; CREBBP; CTDSPL2; CUX2; DDIT4; DLGAP2; DMD; DUSP3; EFNB2; EPC1; EPHA4; FBXL3; FEM1C; FLRT2; FOXO1; FURIN; GALNT7; GLCCI1; HEY2; HLCS; ITSN2; KBTBD8; KLF13; KLF5; KLHL3; LRP12; MAGI1; MAPK4; MCL1; MFAP3L; MFN1; MIER3; MKX; MORC3; NAV2; NFE2L2; NPTN; OTUD4; PALM2; PDS5A; PIK3R1; PPARGC1A; PPM1D; PRDM2; PRR16; PTCH1; QKI; RAI14; RASA1; RGS7BP; RNF165; ROCK2; SEPT11; SGK3; SH3BP4; SLC4A4; SLC9A6; SUN2; TESK2; TGFBR2; TNRC6B; TP53INP1; TP53INP2; UBE2W; UBR1; USP28; WIPF2; ZCCHC14; ZCCHC2; ZFPM2; ZFYVE9; ZNF518A; ZNF609; ZNFX1; ZNRF2 |
| AGCACTT,MIR-93,MIR-302A,MIR-302B,MIR-302C,MIR-302D,MIR-372,MIR-373,MIR-520E,MIR-520A,MIR-526B,MIR-520B,MIR-520C,MIR-520D | ABCA1; ANKRD13C; ANO6; APP; ARHGEF10; ARID4A; ARID4B; ATP2B2; BCL2L11; BRMS1L; C16orf72; CELF2; CFL2; CRIM1; CRK; DAZAP2; DCAF6; DCUN1D1; DDHD1; DHX40; DMTF1; DNAJA2; DNAJC27; DRD1; E2F7; EDNRB; EPAS1; EPHA2; ESR1; FAM13C; FBXO11; FEM1C; FGD5; FLT1; FNDC3A; FOXJ2; GATAD2B; GNB5; INO80; IQSEC2; IRF2; ITGB8; JAZF1; JRKL; KAT2B; KBTBD8; KCNMA1; KLF13; KLHL3; LATS2; LHX6; LUC7L2; MAML1; MARCH8; MBNL2; MCL1; MED12L; MEF2C; MFAP3L; MIER3; MINK1; MTUS1; NAPEPLD; NCOA7; NEK9; NEO1; NFIB; NR2C2; NR4A2; NR4A3; NTN4; NUFIP2; OPCML; OSBPL5; OSBPL8; OTUD4; OXR1; PAN3; PAPOLA; PARP8; PBX3; PCDHA12; PCDHA13; PCDHAC1; PCDHAC2; PGBD5; PHF2; PHF6; PHLPP2; PLEKHA3; PLEKHM1; PPP3R1; PRR16; PRRG1; PURA; RAB11FIP1; RAB22A; RAB6A; RAB6C; RABEP1; RAD23B; RASSF2; RB1CC1; RBL1; REEP3; RGL1; RIC8B; RNF6; RPS6KA1; RPS6KA3; RPS6KA5; RSBN1; RSRC2; SAR1B; SDC1; SEC62; SENP1; SIK1; SIPA1L3; SLITRK3; SS18L1; SSX2IP; TAL1; TAOK2; TBC1D8B; TIPARP; TLE4; TNFAIP1; TNKS2; TOB2; TP53INP1; TP53INP2; TRIP11; TRPS1; TSHZ3; TWF1; TXNIP; UBE2J1; UBE2W; UBR3; ULK1; USP42; USP46; WDR37; YPEL2; YTHDF3; ZBTB11; ZBTB4; ZBTB41; ZBTB6; ZDHHC17; ZFP91; ZFPM2; ZFYVE26; ZKSCAN1; ZMYND11; ZNF436; ZNF654; ZNF800; ZNFX1 |
| ACATTCC,MIR-1,MIR-206 | ABCB7; ACAP2; ADAR; AKAP11; AKAP12; ANKRD29; AP1G1; ARCN1; ARID2; ARRDC3; ATP6V1A; ATP7A; BACH2; BAG4; BICD1; BSDC1; C2orf69; C5orf51; CBL; CCDC141; CDK14; CITED2; CLCN3; CLTC; CNN3; COL4A3; CREBL2; CTTNBP2NL; DAAM1; DDX5; DHX15; DLG4; DNAJC13; EAF1; EFNB2; ETS1; FAM91A1; FBXW7; FGF14; FN1; FNDC3A; FOSB; GCH1; GDF6; HIVEP2; HS3ST3B1; IGF1; KIF2A; KLF13; KTN1; LIN7C; LRRC8A; MAP4K3; MAPKBP1; MED1; MEX3C; MON2; MYLK; MYO1E; NADK; NAP1L5; NR4A2; PAFAH1B1; PBRM1; PDIK1L; PDLIM5; PHF6; PHLPP2; PICALM; PREX1; PSD3; PTPRG; PUM2; QKI; RARB; RASA1; RCAN2; RICTOR; RNF13; RNF138; RNF165; RNF38; RNGTT; RSBN1; RSBN1L; RSPO3; SEC22B; SEC62; SEMA6D; SETBP1; SFRP1; SLC25A25; SLC7A2; SMYD4; SP2; SRGAP2; SULF1; SYNJ2; TBC1D15; TFEC; TGFBR3; TMOD2; TNKS2; TPPP; TRIM2; TSPYL4; TTC7B; TWF1; UBE2H; UBN1; UBQLN1; USP33; UST; VAMP4; WIPF2; ZBTB4; ZBTB6; ZFP36L1; ZMAT3; ZNF236; ZNF280D; ZNF800; ZNF827 |
