# Supplementary material for: Iron-sulphur cluster biogenesis factor LYRM4 is a novel prognostic biomarker associated with immune infiltrates in hepatocellular carcinoma
Source: Cancer Cell Int. 2021 Sep 6;21:463. doi: 10.1186/s12935-021-02131-3 (PMC8419973; doi:10.1186/s12935-021-02131-3)
Supplement: Supplementary file 13 — Additional file 13: Table S9. Significantly enriched transcription factor-target networks of LYRM4 in LIHC (LinkedOmics). [file 12935_2021_2131_MOESM13_ESM.docx]

**Additional file 13: Table S9.** Significantly enriched transcription factor-target networks of *LYRM4* in LIHC (LinkedOmics).

| **Geneset** | **Leading Edge Gene** |
| --- | --- |
| GGAANCGGAANY_UNKNOWN | ATP6V1E1; BANF1; BMS1; CCDC71; CHMP2A; COMMD6; COX6B1; COX7A2; CSNK2B; DLX4; E2F4; EBNA1BP2; EIF1AD; EIF2S3; EIF3H; EIF3K; EPN1; FARSA; GGA1; MRPL21; MRPL43; MRPS18A; MRPS21; MRPS23; NCBP2; PDAP1; POMP; PSMB4; RARS; RNF25; RPL28; RPL38; RUVBL2; SDF2; SEC11A; SEC61G; SMUG1; SNRPE; TAF10; TRIM39; UBA52; UBL5; UBXN1; VPS16 |
| SCGGAAGY_V$ELK1_02 | AATF; AIP; AKT1S1; ANKS3; AP2S1; AP4M1; APTX; ARFGAP1; ARFIP2; ARMC7; ARPC2; ARPC4; ASB1; ASB6; B3GALT6; B3GAT3; BAD; BANF1; BZW2; C12orf57; C14orf119; C19orf47; C1QBP; C6orf47; C6orf89; CBX8; CCDC71; CCDC85B; CCT7; CD2BP2; CDC123; CDC37; CDCA3; CHMP2A; CIAO1; CKS1B; CLN3; CNPY3; COMMD5; COMMD6; COPE; COPS7B; COX17; COX5B; COX6A1; COX6B1; COX8A; CPSF3; CSNK2B; CTNNBL1; CUTC; CWC15; CXXC1; DDX49; DDX50; DGUOK; DIABLO; DLX4; DNAJC7; DNTTIP1; DPCD; E2F4; EBNA1BP2; EEF1B2; EEFSEC; EFTUD2; EIF1AD; EIF3H; EIF3L; EIF4A1; EIF5A; EME1; EMG1; EPN1; ERCC1; ERH; EXOSC3; EXOSC5; FBXL6; FBXW9; FIBP; FKBPL; GAPDH; GAR1; GGA1; GPN2; GRWD1; GTF2A2; HARS; HIF1AN; HSP90AB1; ING4; INO80B; INO80E; IPO4; ITGB1BP1; ITPA; JAGN1; KIF9; KLHDC3; KRTCAP2; LLPH; LMAN2; LSM4; LSM5; LYPLA2; MAD2L1BP; MCM7; MCRS1; MEA1; MED30; MEF2B; METTL5; METTL6; MFSD5; MORN2; MOSPD3; MPDU1; MRM1; MRPL27; MRPL33; MRPL40; MRPL43; MRPL52; MRPS10; MRPS18A; MRPS21; MTMR14; MYL6B; NAGK; NDUFAF3; NEDD8; NKIRAS2; NOC2L; NOL12; NOL7; NOSIP; NPRL2; NTHL1; NUDC; NUDT5; NUP37; NUTF2; OTUB1; OVCA2; PABPC1; PARL; PCGF1; PDAP1; PDCD6; PDLIM1; PEX16; PFDN6; PHF5A; PIGC; POLL; POLR1C; POLR2F; POLR2H; POLR2K; POLR3H; POMP; PPAN; PPIL1; PPP1R11; PPP4C; PRDX5; PRELID1; PRPF19; PSMA4; PSMA6; PSMB1; PSMB7; PSMC1; PSMC4; PSMD13; PTRH2; PUF60; PUS1; RAB24; RABGEF1; RABGGTA; RAD23A; RBCK1; RBM22; RFXANK; RING1; RNF181; RNPS1; ROMO1; RPL11; RPL19; RPL26; RPL27; RPL31; RPL32; RPL36AL; RPL37; RPL37A; RPL6; RPLP2; RPS14; RPS18; RPS19BP1; RPS25; RPS3; RPS3A; RPS5; RPS6; RRS1; RXRB; SAR1A; SART1; SCAMP2; SDF2; SDHAF2; SEC11A; SEC13; SF3B4; SH3GLB2; SIPA1; SIRT3; SIRT6; SLC35C2; SLC39A7; SMUG1; SNF8; SNRPB; SNRPE; SPATA17; SPSB2; SRP14; SRP19; SSBP1; SSU72; STARD3; STOML2; STX10; STX4; SUGT1; SUMO1; SUPT5H; TADA3; TAF10; TAF11; TBC1D13; TBCC; TCOF1; THAP11; THG1L; TIMM10; TMEM101; TMEM199; TMEM208; TMEM222; TMUB1; TOMM20; TOMM22; TOMM40; TRADD; TRAPPC1; TRAPPC4; TRIM11; TRIM39; TRMT1; TRMT112; TRMT6; TRPT1; UBE2E3; UBE2F; UBE2Z; UBL5; UBOX5; UBXN1; UFC1; UQCRH; URM1; USE1; USF1; UXT; VPS16; VPS52; WDR34; WDR46; WDR73; WDR74; WDR83; WFDC3; WRAP53; XAB2; XPO5; YKT6; YWHAE; ZBTB8OS; ZBTB9; ZCCHC7; ZNF394; ZNF408; ZNF580; ZNF668 |
| V$ISRE_01 | ADAM15; AGXT2; AKAP6; AMMECR1; APOOL; ARHGEF6; ARPP21; ASPA; B2M; BBX; BLNK; BTAF1; C5orf51; COL12A1; CREBZF; CRK; CRY1; CXXC5; CXorf21; DDX60; DHX58; DTX3L; DUSP10; EGR2; EPSTI1; ERC1; ERG; ESR1; ETNK1; FBXO11; FMR1; FYCO1; GATA6; GNB4; GRIPAP1; HMCN1; IFI44; IFIH1; IFIT2; IFIT3; IKZF2; IKZF3; IL27; IRF2; KCNN3; KDM4A; KPNA3; LCOR; MED13; NPR3; NUFIP2; OSBP; PCGF5; PGK1; PIGR; PIK3R3; PKN2; PLXNC1; PPARGC1A; PRKACA; PURA; SEMA6D; SH2B3; SH3BGRL; SIK3; SLC24A1; SORBS1; STAT6; TIFA; TOP1; TRIM21; TSC22D2; UBA7; UNC5C; USP18; XAF1; ZEB2; ZFPM1 |
| V$PAX4_02 | AP1G1; ARHGAP24; ARL5B; ATM; ATP2A2; AUTS2; B2M; C5; CAST; CGN; CHD2; CPEB4; CTAGE1; CXXC5; DCX; DOCK11; EBF2; ELK3; EOMES; GGCX; HABP2; IKZF2; IKZF3; ITGA7; KLF13; LCOR; LDB2; LRP5; MAN1C1; MBNL2; MIER3; MITF; MLLT10; MTMR12; MTMR6; NAALADL2; NFIA; NNAT; NPAT; PCDH18; PDZD2; PDZRN4; PITPNM2; PLEKHA6; PTGFR; PTPN21; PTPRG; RAB6A; RAPH1; RNF128; RORA; SGCD; SLC4A4; SOX5; STAT3; SYTL2; TBC1D8B; TCF4; TECTA; TFEC; TGFBR1; TMEM154; TOB1; TSC22D3; USP32; ZFHX4; ZMIZ1 |
| YNGTTNNNATT_UNKNOWN | ACADSB; AMFR; AR; ARL6IP1; ASPA; ATP2A2; BCL2; CALD1; CBLB; CHD2; CHD6; CHMP2B; CITED2; CLMN; CPEB4; CTNND1; DAAM1; DCUN1D1; DIS3L; DLC1; DMD; DOCK4; DUSP10; ELOVL6; EMP1; EOMES; FAM120A; FMR1; GARNL3; GLT8D2; GNAO1; HIPK1; IFIH1; IFT81; IKZF2; ITGA1; ITPR1; JMJD1C; KCNJ2; KDM6A; KIF13A; KLF9; LHX2; LHX6; LRRC4; MAGI1; MAML3; MIA2; MITF; MYOCD; NECAP1; NEDD4; NFIB; NIPBL; NOTCH2; NUMB; OMD; OSBPL9; PAN2; PDE1C; PDZRN4; PGRMC1; PHF6; PIK3R1; PPP2R5E; PRDM1; PRR11; PTPRD; R3HDM2; RAPH1; RBM47; ROR1; RORB; RSF1; RUNX1T1; S100PBP; S1PR1; SATB1; SEC24D; SH2D4A; SLC33A1; SLC5A3; SMARCA2; SMURF2; SORBS2; SOX5; SP4; STAG2; SYTL2; TAB2; TAL1; TCF12; TFEC; THRB; THSD4; TMTC2; TOB1; TPCN2; TRIM68; TSC22D2; TSHZ3; WDTC1; ZBTB37; ZFPM2; ZNF148; ZNF521; ZNF638; ZNF641 |
