# Supplementary material for: Iron-sulphur cluster biogenesis factor LYRM4 is a novel prognostic biomarker associated with immune infiltrates in hepatocellular carcinoma
Source: Cancer Cell Int. 2021 Sep 6;21:463. doi: 10.1186/s12935-021-02131-3 (PMC8419973; doi:10.1186/s12935-021-02131-3)
Supplement: Supplementary file 15 — Additional file 15: Figure S17. PPI network of transcription factor PAX4-target networks (GeneMANIA database). [file 12935_2021_2131_MOESM15_ESM.docx]

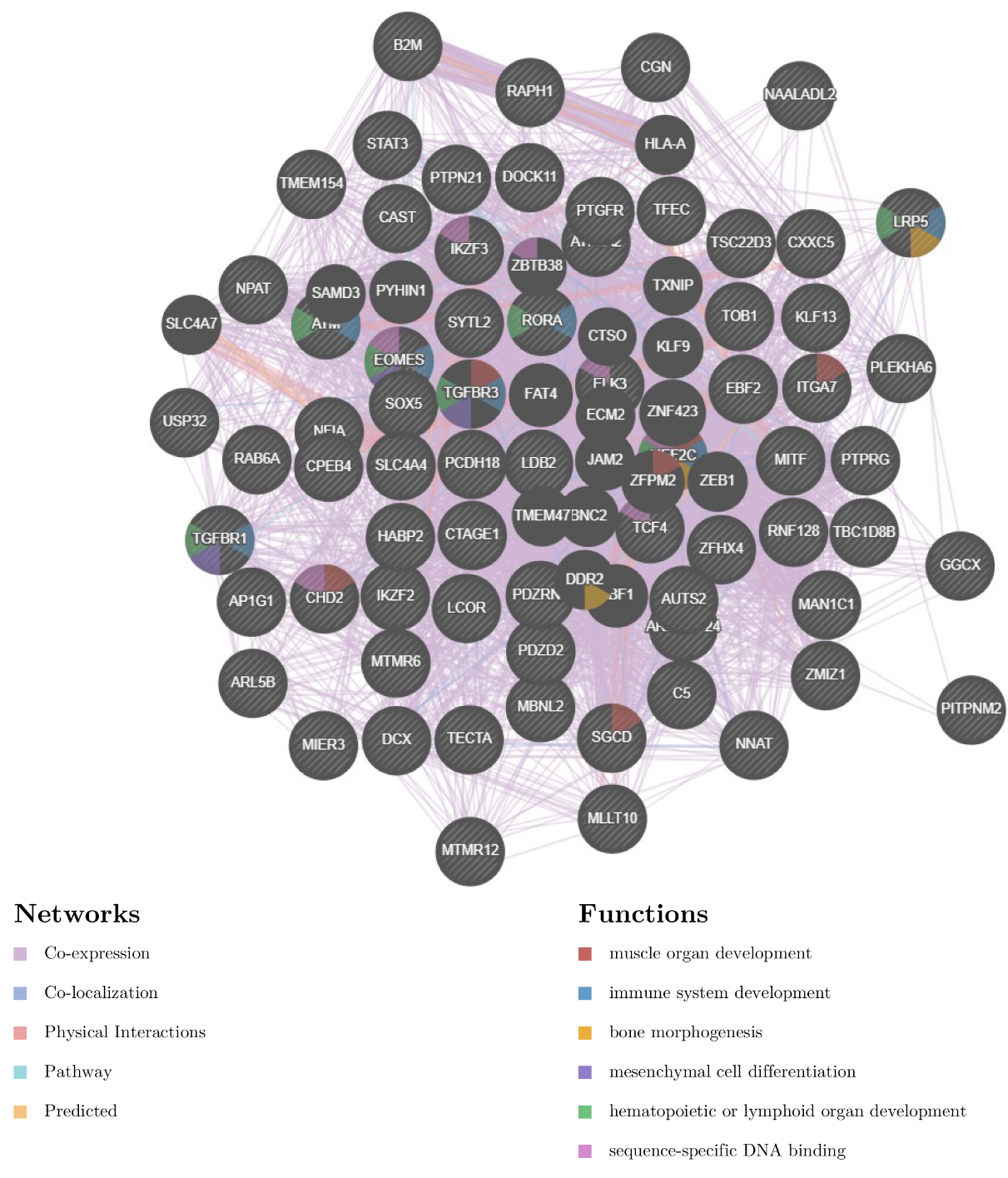


**Additional file 15: Figure S17.** PPI network of transcription factor PAX4-target networks (GeneMANIA database). Colors at network edges indicate the bioinformatics methods applied: co-expression, co-localization, physical interaction, pathway and website prediction. Colors of network nodes indicate the biological functions of enriched gene sets.
