# Supplementary material for: Iron-sulphur cluster biogenesis factor LYRM4 is a novel prognostic biomarker associated with immune infiltrates in hepatocellular carcinoma
Source: Cancer Cell Int. 2021 Sep 6;21:463. doi: 10.1186/s12935-021-02131-3 (PMC8419973; doi:10.1186/s12935-021-02131-3)
Supplement: Supplementary file 16 — Additional file 16: Figure S18. The effect of LYRM4 expression in correlation with the abundance of immune infiltrates on the prognosis of LIHC (TIMER2.0 database). [file 12935_2021_2131_MOESM16_ESM.docx]

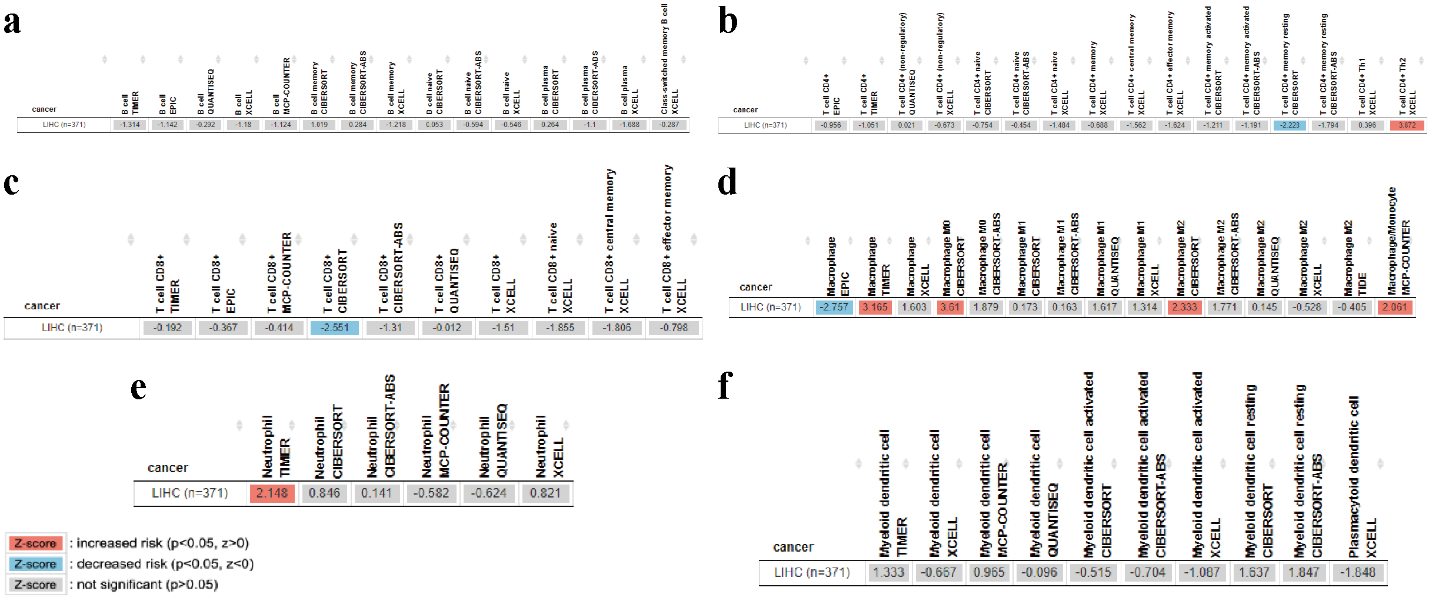


**Additional file 16: Figure S18.** The effect of LYRM4 expression in correlation with the abundance of immune infiltrates on the prognosis of LIHC (TIMER2.0 database). **a-f** Correlation between the different subtypes of immune cell infiltration and LYRM4 expression affects the survival of LIHC patients.
