# Supplementary material for: Iron-sulphur cluster biogenesis factor LYRM4 is a novel prognostic biomarker associated with immune infiltrates in hepatocellular carcinoma
Source: Cancer Cell Int. 2021 Sep 6;21:463. doi: 10.1186/s12935-021-02131-3 (PMC8419973; doi:10.1186/s12935-021-02131-3)
Supplement: Supplementary file 17 — Additional file 17: Figure S19. IscU mRNA expression levels in LIHC. [file 12935_2021_2131_MOESM17_ESM.docx]

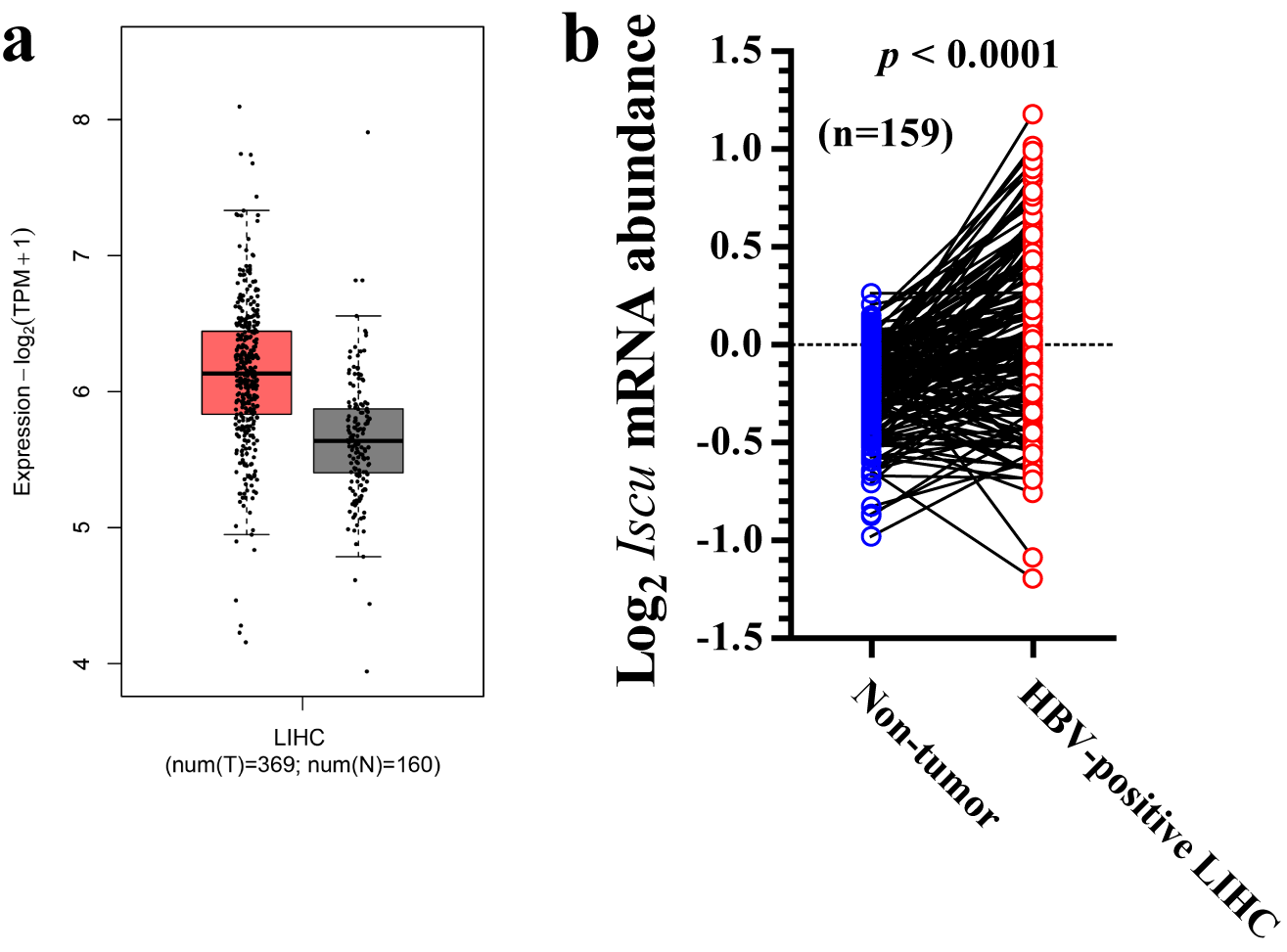


**Additional file 17: Figure S19.** *IscU* mRNA expression levels in LIHC. **a** *IscU* mRNA expression levels in LIHC tissues and adjacent normal liver tissues from GEPIA 2 database. **b** The mRNA abundance of *IscU* in HBV-Related LIHC and paired non-tumor liver tissues was investigated by RNA-seq (n=159). This figure results were obtained from Gao *et al*. [5] studies.
