# Supplementary material for: Iron-sulphur cluster biogenesis factor LYRM4 is a novel prognostic biomarker associated with immune infiltrates in hepatocellular carcinoma
Source: Cancer Cell Int. 2021 Sep 6;21:463. doi: 10.1186/s12935-021-02131-3 (PMC8419973; doi:10.1186/s12935-021-02131-3)
Supplement: Supplementary file 3 — Additional file 3: Figure S2–13. Original data. [file 12935_2021_2131_MOESM3_ESM.doc]

Original data

**Additional file 3: Figure S2**, related to Figure 5b. Representation of original immunohistochemistry images with different magnification of LYRM4 in human kidney tissue (as positive control).

**Additional file 3: Figure S3**, related to Figure 5b-c. Representative original immunohistochemistry images with different magnification of LYRM4 in normal liver tissue.

**Additional file 3: Figure S4**, related to Figure 5b-c. Representative original immunohistochemistry images with different magnification of LYRM4 in 92 matched LIHC tissues.

**Additional file 3: Figure S5**, related to Figure 5d. Expression of LYRM4 and related iron-sulphur protein in three LIHC cell lines and hepatocytes by western blotting.

**Additional file 3: Figure S6**, related to Figure 5k. The activity of mitochondrial complex I and citrate synthase (CS) in hepatocytes L-02 were detected by UV-vis spectrophotometry. (A-C) Prepared mitochondria (4 µl; 2 µg/µl) were added to the assay buffer containing potassium phosphate buffer (20 µl, 0.5 M, pH 7.5), fatty acid-free BSA (12 µl, 50 mg/ml), NaN_3_ (2 µl, 0.5 M), Antimycin A (0.4 µl, 1mg/ml), NADH (2 µl, 10 mM), and distilled water (155.6 µl). After reading the baseline at 340 nm for 2 minutes, ubiquinone (4 µl, 6 mM) was applied and the decrease in absorbance was recorded at 340 nm for 90 s. (D-F) In parallel, the same quantity of reagents and samples but with the addition of rotenone solution (2 µl; 1 mM) was used. (G-I) The activity of mitochondrial CS in L-02 cells was measured by spectrophotometry. The assay buffer containing Tris (200 mM, pH8.0) + Triton X-100 (0.2%) (100 µl), DTNB (20 µl, 1 mM), acetyl coenzyme A (6 µl, 10 mM), and distilled water (63 µl) incubated at 37℃ for 5 min, oxaloacetate (10 µl, 10 mM) and prepared mitochondria protein (1 µl, 2 µg/µl) were added and then the increase in absorbance at 412 nm was recorded for 1 minutes.

**Additional file 3: Figure S7**, related to Figure 5k. The activity of mitochondrial complex I and CS in LIHC cell lines HepG2 was detected by UV-vis spectrophotometry. (A-C) Activity of complex I in mitochondria of HepG2 cells was detected by spectrophotometry, and the complex I inhibitor rotenone was not added. (D-F) In parallel, the same quantity of reagents and samples but with the addition of rotenone solution (2 µl; 1 mM) was used. (G-I) The activity of mitochondrial CS in HepG2 cells was measured by spectrophotometry.

**Additional file 3: Figure S8**, related to Figure 5k. The activity of mitochondrial complex I and CS in LIHC cell lines HCCLM3 was detected by UV-vis spectrophotometry. (A-C) Activity of complex I in mitochondria of HCCLM3 cells was detected by spectrophotometry, and the complex I inhibitor rotenone was not added. (D-F) In parallel, the same quantity of reagents and samples but with the addition of rotenone solution (2 µl; 1 mM) was used. (G-I) The activity of mitochondrial CS in HCCLM3 cells was measured by spectrophotometry.

**Additional file 3: Figure S9**, related to Figure 5k. The activity of mitochondrial complex I and CS in LIHC cell lines MHCC97H was detected by UV-vis spectrophotometry. (A-C) Activity of complex I in mitochondria of MHCC97H cells was detected by spectrophotometry, and the complex I inhibitor rotenone was not added. (D-F) In parallel, the same quantity of reagents and samples but with the addition of rotenone solution (2 µl; 1 mM) was used. (G-I) The activity of mitochondrial CS in MHCC97H cells was measured by spectrophotometry.

**Additional file 3: Figure S10**, related to Figure 5l. The activity of mitochondrial ACO2 and citrate synthase (CS) in human hepatocytes L-02 were detected by UV-vis spectrophotometry. (A-C) Activity of ACO2 of L-02 cells was detected by spectrophotometry. Add the prepared mitochondria (5 µl; 2 µg/µl) to the assay buffer containing Tris pH8.0 (100 mM), cis-aconitic acid (1 mM), MgCl_2_ (5 mM) and isocitrate dehydrogenase (4 U/ml). Incubated at 37℃ for 2 min. After reading the baseline at 340 nm, adding NADP^+^ and record the increase in absorbance at 340 nm for 80 s. (D-F) The activity of CS in L-02 cells was measured by spectrophotometry. The enzymatic activity of each mitochondrial enzyme should be corrected by CS activity.

**Additional file 3: Figure S11**, related to Figure 5l. The activity of ACO2 and CS in human LIHC cell lines HepG2 were detected by UV-vis spectrophotometry. (A-C) Activity of ACO2 of HepG2 cells was detected by spectrophotometry. (D-F) The activity of CS in HepG2 cells was measured by spectrophotometry.

**Additional file 3: Figure S12**, related to Figure 5l. The activity of ACO2 and CS in human LIHC cell lines HCCLM3 were detected by UV-vis spectrophotometry. (A-C) Activity of ACO2 of HCCLM3 cells was detected by spectrophotometry. (D-F) The activity of CS in HCCLM3 cells was measured by spectrophotometry.

**Additional file 3: Figure S13**, related to Figure 5l. The activity of ACO2 and CS in human LIHC cell lines MHCC97H were detected by UV-vis spectrophotometry. (A-C) Activity of ACO2 of MHCC97H cells was detected by spectrophotometry. (D-F) The activity of CS in MHCC97H cells was measured by spectrophotometry.
